# Direct and plant‐mediated effects of climate on bird diversity in tropical mountains

**DOI:** 10.1002/ece3.7014

**Published:** 2020-11-13

**Authors:** Maximilian G. R. Vollstädt, Jörg Albrecht, Katrin Böhning‐Gaese, Andreas Hemp, Kim M. Howell, Laura Kettering, Alexander Neu, Eike Lena Neuschulz, Marta Quitián, Vinicio E. Santillán, Till Töpfer, Matthias Schleuning, Susanne A. Fritz

**Affiliations:** ^1^ Senckenberg Biodiversity and Climate Research Centre (SBiK‐F) Frankfurt am Main Germany; ^2^ Institute for Ecology, Evolution and Diversity Goethe University Frankfurt am Main Germany; ^3^ Department of Plant Systematics University of Bayreuth Bayreuth Germany; ^4^ Department of Zoology and Wildlife Conservation University of Dar es Salaam Dar es Salaam Tanzania; ^5^ Zoological Research Museum Alexander Koenig Bonn Germany

**Keywords:** Andes, fruiting plants, functional diversity, intercontinental comparison, Mt. Kilimanjaro, resource effects

## Abstract

**Aim:**

Although patterns of biodiversity across the globe are well studied, there is still a controversial debate about the underlying mechanisms and their generality across biogeographic scales. In particular, it is unclear to what extent diversity patterns along environmental gradients are directly driven by abiotic factors, such as climate, or indirectly mediated through biotic factors, such as resource effects on consumers.

**Location:**

Andes, Southern Ecuador; Mt. Kilimanjaro, Tanzania.

**Methods:**

We studied the diversity of fleshy‐fruited plants and avian frugivores at the taxonomic level, that is, species richness and abundance, as well as at the level of functional traits, that is, functional richness and functional dispersion. We compared two important biodiversity hotspots in mountain systems of the Neotropics and Afrotropics. We used field data of plant and bird communities, including trait measurements of 367 plant and bird species. Using structural equation modeling, we disentangled direct and indirect effects of climate and the diversity of plant communities on the diversity of bird communities.

**Results:**

We found significant bottom‐up effects of fruit diversity on frugivore diversity at the taxonomic level. In contrast, climate was more important for patterns of functional diversity, with plant communities being mostly related to precipitation, and bird communities being most strongly related to temperature.

**Main conclusions:**

Our results illustrate the general importance of bottom‐up mechanisms for the taxonomic diversity of consumers, suggesting the importance of active resource tracking. Our results also suggest that it might be difficult to identify signals of ecological fitting between functional plant and animal traits across biogeographic regions, since different species groups may respond to different climatic drivers. This decoupling between resource and consumer communities could increase under future climate change if plant and animal communities are consistently related to distinct climatic drivers.

## Graphical abstract

Although patterns of biodiversity across the globe are well studied, there is still a controversial debate about the underlying mechanisms and their generality across biogeographic scales. We found significant bottom‐up effects of fruit diversity on frugivore diversity at the taxonomic level. In contrast, climate was more important for patterns of functional diversity, with plant communities being mostly related to precipitation, and bird communities being most strongly related to temperature.

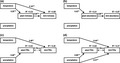

## 1 INTRODUCTION

The distribution of biodiversity on earth has fascinated scientists for over two centuries (e.g., Darwin, 1859; von Humboldt, 1808). Consequently, the patterns of biodiversity along spatial and environmental gradients are very well documented (Gaston, 2000). Biodiversity generally peaks in the tropics and decreases toward higher latitudes (Allen et al., 2002; Hillebrand, 2004) and additionally decreases along elevational gradients in mountain systems (e.g., Rahbek, 1995). Despite the extensive knowledge on biodiversity and its distribution, understanding the mechanisms behind these patterns still constitutes a major challenge (Lewinsohn & Roslin, 2008; Mittelbach et al., 2007). For instance, many studies argue for taking the historical background of a biogeographical region into account to capture differences in colonization times or net diversification rates among continents and biogeographic realms (e.g., Jetz & Fine, 2012; Wiens & Donoghue, 2004). Furthermore, a large body of evidence links species coexistence and the resulting patterns of species diversity to present‐day abiotic and biotic drivers, such as energy availability (Guégan et al., 1998; Rosenzweig, 1992; Roy et al., 1998) or biotic interactions (Bascompte, 2009).

Despite the controversial discussion about the mechanisms behind spatial biodiversity patterns, it is widely recognized that climatic conditions are fundamentally correlated with biodiversity (Evans et al., 2005). For instance, warm and humid regions usually support higher levels of biodiversity than cold and dry regions because of the physiological limitations imposed by temperature and water availability on species’ occurrence (Currie et al., 2004). However, species do not occur alone, but coexist and interact with each other in ecological communities (e.g., Bascompte, 2009). It has been shown that the diversity of species from higher trophic levels, that is, consumers, can depend on the diversity of species from lower trophic levels, that is, resources, because of bottom‐up effects (Albrecht et al., 2014; Hanz et al., 2019; Scherber et al., 2010). While top‐down regulation may also occur, such processes seem to be more important in complex systems with long trophic chains (e.g., Sandom et al., 2013). Bottom‐up mechanisms are especially important in systems with short trophic chains, such as seed‐dispersal systems (Albrecht et al., 2014; Ferger et al., 2014; Vollstädt et al., 2017). If bottom‐up mechanisms prevail, the diversity of primary producers is expected to be directly driven by energy availability, leading to direct climatic effects on the diversity of lower trophic levels and to indirect climatic effects on higher trophic levels (Wright, 1983). Yet, studies simultaneously disentangling direct and indirect effects of climate and resources on biodiversity remain scarce (but see Ferger et al., 2014; Vollstädt et al., 2017), especially across biogeographic regions (but see Hanz et al., 2019) and trophic levels. Specifically, our knowledge of bottom‐up effects on avian seed‐dispersal communities across different biogeographic regions is limited.

Bottom‐up effects of resources can potentially drive the diversity of species from higher trophic levels through different mechanisms. For instance, the species richness of consumers may increase with higher abundance of resources (e.g., Ferger et al., 2014; Kissling et al., 2007) and may additionally follow patterns of fluctuating resource availability (Mulwa et al., 2012). Such patterns suggest the necessity for behavioral flexibility of consumers, since they need to actively track specific resources and their availability (Herrera, 1984, 1985).

Further, morphological barriers between species from different trophic levels can present a limiting factor. When species from different trophic levels interact, such as in seed‐dispersal interactions, they depend on specific functional traits that facilitate the matching of interaction partners (Garibaldi et al., 2015; Jordano, 1987). For instance, bill size of birds limits the size of fruit, which avian frugivores may successfully handle (Dehling et al., 2014; Wheelwright, 1985). Consistent with the concept of ecological fitting (Janzen, 1985), it was recently shown how the diversity of functional traits of fruit resources can result in a bottom‐up regulation of the corresponding functional diversity of avian consumers across large environmental gradients, suggesting trait matching between species groups (Dehling et al., 2014; Quitián et al., 2019; Vollstädt et al., 2017). Such bottom‐up effects mediated by species traits can be described by the size of the trait space of those functional traits that are important for interactions, such as seed‐dispersal interactions between plants and birds.

The distribution of consumer species in a functional trait space can reflect different patterns of niche occupancy (e.g., Pigot et al., 2016). For instance, functional dispersion has been associated with the expansion of a community's trait space because competition for limited resources may lead to species with more specialized functional traits (Karr & James, 1975; Macarthur, 1965). In contrast, functional clustering may occur, when environmental filtering leads to functionally similar species, which densely fill the community trait space (Karr & James, 1975; Klopfer & Macarthur, 1961). However, our knowledge about the relative importance of direct abiotic and indirect biotic effects on the patterns of niche occupancy across large environmental gradients is still very limited.

Seed dispersal by avian frugivores is ideally suited to study direct abiotic and indirect bottom‐up effects of lower trophic levels on the taxonomic and functional diversity of higher trophic levels across environmental gradients (Vollstädt et al., 2017). Fruit resources fluctuate in their spatiotemporal availability, and specific plant and animal traits, such as fruit size in plants and bill shape in birds, are key matching traits that set the blueprint for pair‐wise species interactions (Dehling et al., 2014; Wheelwright, 1985). Moreover, tropical mountains are ideally suited for studies of covariation in species communities across environmental gradients, since the climatic conditions vary strongly over comparatively small spatial extents (Sanders & Rahbek, 2012). As diversity of frugivores and plants varies fundamentally among biogeographic regions (Fleming et al., 1987; Kissling et al., 2012; Qian & Ricklefs, 2007), we perform the first intercontinental comparison to test for the combined effects of climate and resources on frugivore diversity in two regions with different biogeographic history.

**FIGURE 1:**
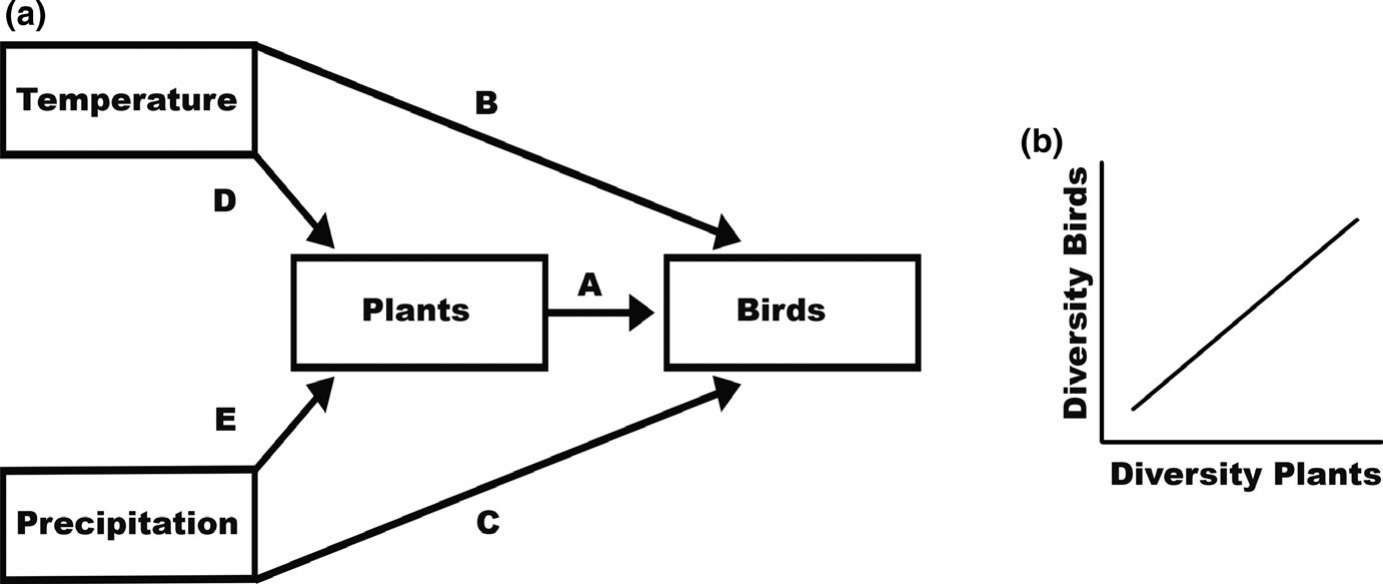
(a) A priori hypothesized causal structure of the relationships between bird communities, plant resources, and climate (temperature and precipitation). The possible links are numbered alphabetically. The relative importance of each link in the models can be seen in Table 1. (b) Direct relationship between bird and plant communities at the level of taxonomic, as well as functional diversity (link A)

Here, we investigate patterns of species diversity of interdependent species groups at both the taxonomic level, that is, richness and abundance, and the level of functional traits that are relevant for seed‐dispersal interactions. We compare these patterns across large environmental gradients between two major biodiversity hotspots of the Neotropics and the Afrotropics. We use field data of plant and bird communities, including trait measurements of 367 plant and bird species, from the Andes of southern Ecuador and from Mt. Kilimanjaro in Tanzania. Using structural equation models, we simultaneously test the effects of climate as well as the effects of resources on the diversity of frugivorous bird species. Specifically, we test the following hypotheses:

First, if bottom‐up mechanisms play an important role in shaping the diversity of higher trophic levels, we should find strong links between the taxonomic diversity of fruiting plants and frugivorous birds along both elevational gradients (link A in Figure 1a, Figure 1b). Similarly, if trait matching is an important mechanism that influences interactions between resources and consumers, we should find a positive link between the functional diversity of fruiting plants and frugivorous birds along both elevational gradients (link A in Figure 1a, Figure 1b). Second, if bottom‐up mechanisms of fruit resources are important for the regulation of the diversity of frugivorous birds, the direct relationships of climate with taxonomic and functional diversity of frugivorous birds should be weak (links B, C in Figure 1a). Instead, these relationships should be indirectly mediated through the taxonomic and functional diversity of plants (links D, E + A in Figure 1a).

## 2 METHODS

### 2.1 Study area

The study was conducted in Podocarpus National Park in the Eastern Cordillera of the southern Ecuadorian Andes (3°58′–4°6′S, 78°58′–79°10′W, see Appendix S1 in Figure S1.1) and on Mt. Kilimanjaro in northern Tanzania (2°45′–3°25′S, 37°0′–37°43′E, see Appendix S1, Figure S1.2). Temperature decreases linearly with increasing elevation at both locations (Hemp, 2005; Moser et al., 2007). Rainfall increases with increasing elevation in Podocarpus National Park (Moser et al., 2007), while it peaks at an elevation of about 2,200 m asl on Mt. Kilimanjaro, decreasing toward lower and higher elevations, respectively (Hemp, 2005). In total, we combined data from 38 study plots (18 plots in the Ecuadorian Andes, 20 plots on Mt. Kilimanjaro). In the Ecuadorian Andes, we collected data on six replicate study plots of 30 × 100 m in three habitat types covering three elevations, respectively: evergreen premontane forest (1,000 m asl), lower montane forest (2,000 m asl) and upper montane forest (3,000 m asl). The forest within Podocarpus National Park is considered mostly undisturbed (Homeier et al., 2008). On Mt. Kilimanjaro, we collected data on five replicate study plots of 30 × 100 m in four undisturbed habitat types at four elevations, respectively: colline savanna (870–1,150 m asl), lower montane forest, (1,620–2,020 m asl), montane *Ocotea* forest (2,120–2,750 m asl) and upper montane *Podocarpus* forest (2,720–3,060 m asl).

### 2.2 Climate

To describe the climatic conditions along the two elevational gradients, we compiled plot‐level data on the mean annual temperature and mean annual precipitation available for both study regions. Plot‐level data are especially important in tropical mountain systems, as regional and global datasets are interpolated, resulting in low precision of measurements in montane environments. In the Ecuadorian Andes, the data were collected through an air temperature regionalization tool, which was designed for the study region (Fries et al., 2009) and through a combination of remote sensing and meteorological data (Rollenbeck & Bendix, 2011). This combination is best suited to derive local climate information for the southern slopes of the Ecuadorian Andes (Fries et al., 2009; Rollenbeck, 2006). On Mt. Kilimanjaro, data were collected over 15 years through a network of temperature loggers (maximum – minimum thermometers; ±0.1°C) and rain gauges (dipping bucket and funnel gauges, ±1 mm) distributed across the mountain (Hemp, 2006).

### 2.3 Plant and bird communities

In the Andes, we recorded plant and bird communities between 2014 and 2015, twice during the wettest months (May–July) and twice during the driest months (October–December), resulting in four temporal replicates for each habitat type (Quitián et al., 2018; Santillán et al., 2018). On Mt Kilimanjaro, we recorded communities between 2013 and 2015, twice during the cold dry season (June–September) and twice during the warm dry season (January–February), resulting in four temporal replicates for each habitat type (Vollstädt et al., 2017, 2018).

We recorded the communities of fleshy‐fruited plants and frugivorous birds on each plot in both mountain ranges. Each plot covered a representative amount of fruiting plants typical for each habitat type. We recorded, mapped and identified all fruiting plants to species level. To assess resource abundance, we estimated the total number of ripe fruits for each plant individual. On plants with very large crop sizes, we counted the number of fruits for representative branches and used these to estimate the crop size of the whole plant.

We recorded the communities of frugivorous birds by observing interactions with fruiting plants using binoculars. The entire area of the plot was observed with equal attention with the aid of field assistants. On each plot, in each of the seasonal samples, frugivores were observed for a total of 25 hr distributed over five consecutive days (Quitián et al., 2018; Vollstädt et al., 2017, 2018). Birds were identified using Ridgely and Greenfield (2001) in the Ecuadorian Andes and using Zimmerman et al. (1999) on Mt. Kilimanjaro. Only interaction events considered as legitimate seed dispersal (i.e., swallowing or transporting seeds away from mother plants) were included in the analysis. Sampling effort was sufficient to cover the pool of frugivorous birds in each respective habitat, as indicated by sampling curves (Ecuadorian Andes: see Figure A1 in Quitián et al., 2017; Mt. Kilimanjaro: see Appendix 7 in Vollstädt et al., 2018).

### 2.4 Functional traits

To quantify the functional diversity of fruiting plant and frugivorous bird communities, we measured plant and bird traits that are known to influence interactions between the two species groups (Dehling et al., 2014). For fruiting plants, we measured four functional traits: fruit length, fruit diameter, plant height and crop mass. In the field, we collected 15 fruits for each plant species (five fruits each from three different individuals). We measured the length of the fruit (peduncle to tip) and the maximum diameter of the fruit using a sliding caliper (±0.01 mm). We measured the height of all plant individuals of each species using a laser range finder (±1 m). We weighed fruits using a digital scale (±0.01 g) and multiplied mean fruit mass with the mean number of fruits per plant species to calculate crop mass for each species.

For frugivorous birds, we also considered four functional traits: bill length, bill width, Kipp's index and body mass. All traits were measured on museum specimens (Natural History Museum, Berlin; Museo Ecuatoriano de Ciencias Naturales, Quito; Zoological Research Museum Alexander Koenig, Bonn; Zoological Museum of Denmark at the University of Copenhagen). We measured two female and two male specimens of each species. We measured bill length and bill width using a sliding caliper (±0.01 mm). We measured bill length as the distance from the commissural point of the upper and lower bill to the tip of the closed bill, and bill width as the external distance between the two commissural points, which is functionally equivalent to gape width (Wheelwright, 1985). We derived Kipp's index from measuring Kipp's distance (distance between tip of the first secondary and tip of the longest primary of the folded wing) as well as wing length; Kipp's index was then calculated as the ratio between Kipp's distance and wing length and is a measure of wing shape. We followed Eck et al. (2011) for all bird measurements. We compiled data on avian body mass using Dunning (2007). Except Kipp's index, all plant and bird traits were log‐transformed to approximate normality.

### 2.5 Taxonomic and functional diversity

We calculated two measures of taxonomic diversity. First, we measured species richness of each plant and bird community as the number of species recorded on each study plot. Second, we measured abundance of species in each plant and bird community. Abundance of plant resources in each community was measured as the sum of the crop sizes of all plant species recorded on each study plot. Abundance of birds in each community was measured as the total number of legitimate seed‐dispersal visits of all frugivore species on each study plot. All abundance measures were log‐transformed prior to analysis.

Additionally, we calculated two multivariate indices of functional trait diversity for plant and bird communities on each study plot: functional richness (FRic; Villéger et al., 2008) and functional dispersion (FDis; Laliberté & Legendre, 2010). Calculation was based on the Euclidean distances between species in a Principal Coordinates Analysis (PCoA) that was used to project species into a multidimensional trait space. Functional richness is a measure of the total morphological variety of a species community and is given by the size of a convex hull around all individual species in multidimensional space. The functional richness value for each community was then standardized against the total functional richness value in each biogeographic region calculated from the mountain‐level regional species pool (i.e., all species recorded on any plot along each elevational gradient). Functional dispersion measures the mean distance of species in a community to the centroid of all species in that community. It is abundance weighted and therefore not as strongly influenced by extreme values as functional richness. For some plots, it was not possible to calculate functional richness values because either the fruiting plant or the avian frugivore communities were too small, that is, less than three species occurred in either trophic group. Overall, we were able to compute functional richness values for 16 plots in the Ecuadorian Andes and 18 plots on Mt. Kilimanjaro, and functional dispersion values for 17 plots in the Ecuadorian Andes and 18 plots on Mt. Kilimanjaro.

To test whether the indices of functional diversity were mostly driven by species richness patterns, we additionally compared observed to randomized communities derived from null models and calculated standardized effect sizes (FRic_SES_ and FDis_SES_). We used the “independent swap” algorithm (Gotelli, 2000) to create 1,000 randomized communities. This algorithm maintains the total occurrence frequency of each species and species richness of each community but randomizes species identity and abundance across communities. This allows to test for the effects of abiotic and biotic variables on functional diversity after accounting for the effects of species richness. Standardized effect sizes were calculated by subtracting the mean of the randomized values for each community from the observed value of that community and dividing the results by the standard deviation of the randomized values.

### 2.6 Statistical analysis

To disentangle effects of climate and taxonomic or functional diversity of plant communities on the taxonomic or functional diversity of bird communities, we used structural equation models (SEMs) which are able to account for both direct and indirect relationships among variables in complex systems (Grace et al., 2012; Sonne et al., 2016). We first defined an a priori structure of the SEM, focusing on bottom‐up mechanisms. This SEM included all biologically plausible links between bird communities, plant communities, temperature and precipitation (Figure 1a). This approach allowed us to compare the respective importance of abiotic versus biotic drivers and their direct and indirect effects on bird communities. In order to understand the general importance of each driver for the bird communities, we ran the analyses across both biogeographic regions (i.e., Ecuadorian Andes and Mt. Kilimanjaro). To this end, we fitted the SEMs with biogeographic region as factor. We did not explicitly test for effects of the respective biogeographic region, because of sample size limitations and because results were qualitatively similar between biogeographic regions.

**FIGURE 2:**
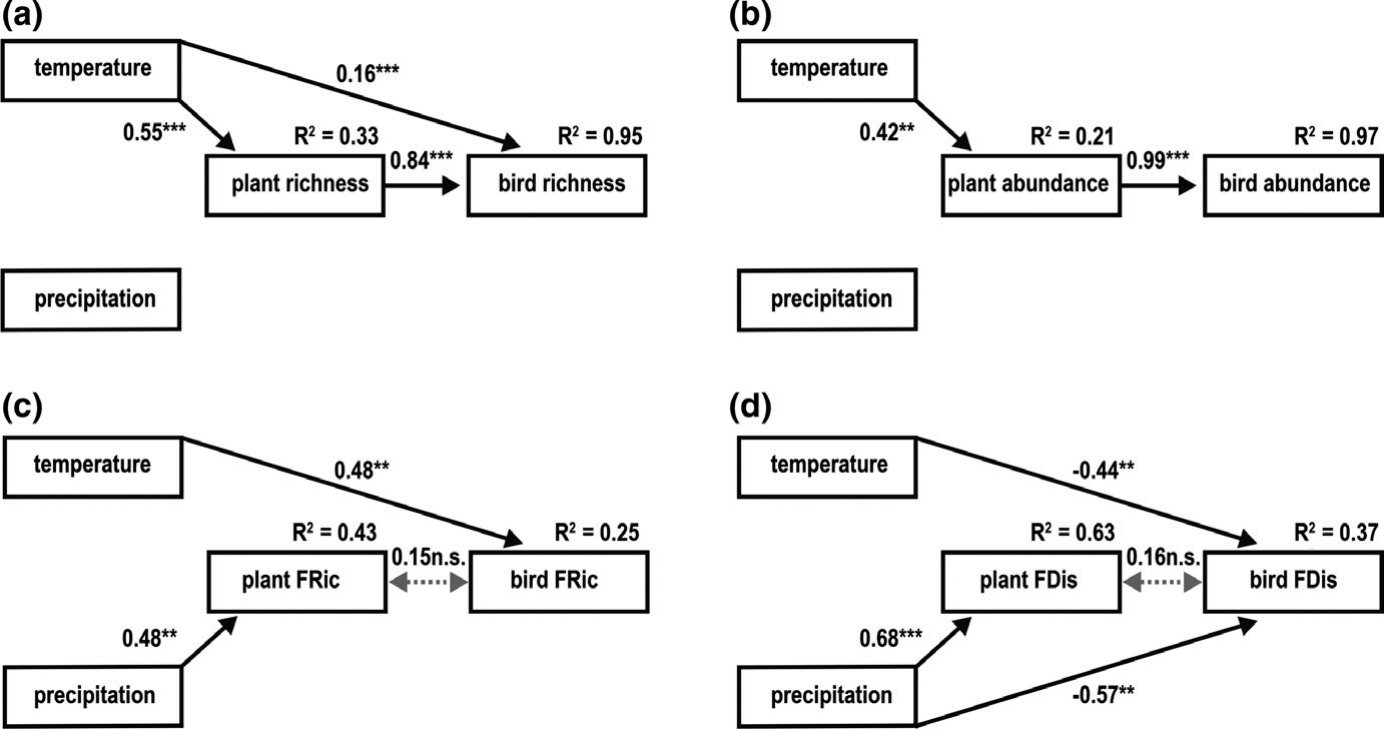
Relationships between bird communities, plant resource communities, and climate (temperature and precipitation) in the Ecuadorian Andes and on Mt. Kilimanjaro, according to the models with highest model fit for (a) species richness, (b) species abundance, (c) functional richness (FRic), and (d) functional dispersion (FDis). The path coefficients for paths in the best‐fit model, their statistical significance (*p < 0.05, **p < 0.01, ***p < 0.001), and the coefficients of determination (*R* ^2^) are given. Nonsignificant paths that were retained in the best models are represented by dotted lines

For each metric of taxonomic and functional diversity (species richness, species abundance, FRic, FDis, FRic_SES_ and FDis_SES_), we fitted a separate SEM. Thus, we considered only corresponding metrics of plant and bird communities in each model (see Figure 2; see Appendix S2, Figure S2.3). We *z*‐transformed all variables to zero mean and unit variance prior to analysis, to allow for comparison of the effect sizes between predictor and response variables with different scales. Based on the full a priori SEM, we performed a stepwise removal of nonsignificant paths to simplify the SEM, which is a widely accepted approach (Sonne et al., 2016). We first excluded paths with the highest *P*‐value and repeated this procedure until we reached the most parsimonious SEM for each response variable, which we identified using the information theoretic approach (i.e., the model characterized by the lowest AICc value) (Shipley, 2013). We evaluated the resulting SEM through a *χ* ^2^ test, the root mean square error of approximation (RMSEA) and the comparative fit index (CFI) (Grace et al., 2012; Hooper et al., 2008). A nonsignificant result of the *χ* ^2^ test (*p* > 0.05, Hooper et al., 2008), lower 90% of confidence intervals of RMSEA close to 0 (Grace et al., 2012) and CFI values larger than 0.95 (Hu & Bentler, 1999) indicate a good fit of the model to the observed data.

We tested the null expectation, that is, no relationship between bird and plant communities, by fitting environment SEMs for each diversity metric, where potential bottom‐up effects of plant communities were excluded (Sandom et al., 2013). The missing path was then accounted for in the model by adding error covariance between variable pairs (Sandom et al., 2013).

**FIGURE 3:**
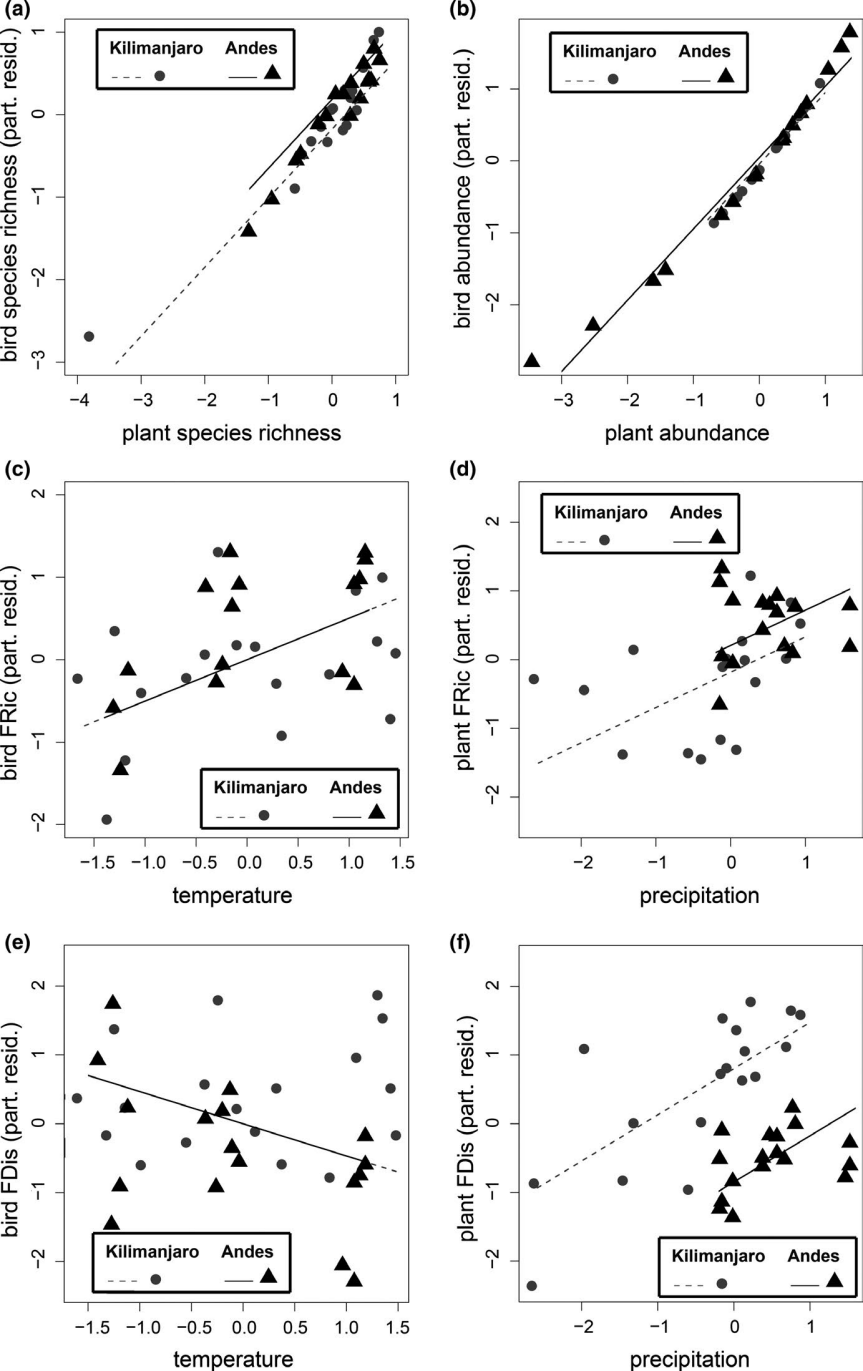
Taxonomic and functional diversity values of bird and plant communities across the most important gradients according to our models in the Ecuadorian Andes and on Mt. Kilimanjaro, for (a) species richness, (b) species abundance, (c) bird functional richness (FRic), (d) plant functional richness (FRic), (e) bird functional dispersion (FDis), and (f) plant functional dispersion (FDis). Shown are the relationships with the predictor variable that had the strongest link with the respective measure of diversity in the best‐fit model (see Table 1, Figure 2). Data points represent partial residuals from this model for each study plot of the respective mountain range. Sample sizes were as follows: N (a) + (b) Ecuadorian Andes = 17, Mt. Kilimanjaro = 18; N (c) + (d) Ecuadorian Andes = 16, Mt. Kilimanjaro = 18; N (e) + (f) Ecuadorian Andes = 17, Mt. Kilimanjaro = 18

To visualize the patterns of taxonomic and functional diversity across the most important gradients according to the most parsimonious SEM, we plotted partial residuals from the respective models (Figure 3).

All statistical analyses were performed with R version 3.5.1 (R Core Team, 2012) and the packages “FD” (Laliberté et al., 2014), “picante” (Kembel et al., 2010) and “lavaan” (Rosseel, 2012).

## 3 RESULTS

Overall, we recorded 112 fruiting plant species and 130 frugivorous bird species across all plots of the Ecuadorian Andes, and 53 fruiting plant species and 72 frugivorous bird species across all plots of Mt. Kilimanjaro (see Appendix S3, Tables S3.3–S3.4). In the Ecuadorian Andes, the dominant, most frequently visited plants at lower elevations were species from the genera *Miconia* (779 visits) and *Cecropia* (492 visits), and *Miconia* (32 visits) was equally among the most frequently visited genera at higher altitudes (see Appendix S3, Table S3.3). The Paradise Tanager (*Tangara chilensis*, 531 visits) was the most frequent bird species at lower elevations of the Ecuadorian Andes, while the Lacrimose Mountain‐Tanager (*Anisognathus lacrymosus*, 60 visits) was most frequent at higher elevations (see Appendix S3, Table S3.4). On Mt. Kilimanjaro, small trees such as *Lannea* (307 visits) and *Ozoroa* (91 visits) were frequently visited at lower elevations, while *Schefflera* (2,573 visits) was most frequently visited by birds at high elevations (see Appendix S3, Table S3.3). The Common Bulbul (*Pycnonotus barbatus,* 980 visits) was the most frequent bird species at low elevations, while the Montane White‐Eye (*Zosterops poliogaster*, 1,926 visits) was the most frequent visitor at high elevations of Mt. Kilimanjaro (see Appendix S3, Table S3.4).

Overall, plant and bird species richness varied from 8–22 (median = 13) and 2–53 (median = 23) species per plot in the Ecuadorian Andes and 5–15 (median = 7.5) and 7–23 (median = 12.5) species per plot on Mt. Kilimanjaro, respectively. Species turn‐over between plots was high and, on average, 90% of plant species in the Ecuadorian Andes and 88% on Mt. Kilimanjaro, and 80% of bird species in the Ecuadorian Andes and 83% on Mt. Kilimanjaro were replaced between plots (Sørensen dissimilarity, mean ± *SD* along each elevational gradient; plants Ecuadorian Andes: 0.90 ± 0.23, Mt. Kilimanjaro: 0.88 ± 0.24; birds Ecuadorian Andes: 0.80 ± 0.27, Mt. Kilimanjaro: 0.83 ± 0.28). Moreover, FD of plant and bird communities varied across both mountain ranges (e.g., plant FRic Ecuadorian Andes: < 0.01–0.08, Mt. Kilimanjaro: < 0.01–0.06; bird FRic Ecuadorian Andes: < 0.01–0.20, Mt. Kilimanjaro: < 0.01–0.20; see Appendix S3, Table S3.2).

### 3.1 Drivers of taxonomic and functional diversity

The best‐fit SEMs for all variables showed a good fit to the data (in all cases: *P*(*χ* ^2^) > 0.05; lower 90% of confidence intervals of RMSEA close to 0; CFI > 0.95). Results were consistent across biogeographic regions, with exception of plant functional dispersion (FDis, see Figure 3a–f).

Species richness of bird communities was strongly related to species richness of plants, which mediated indirect effects of mean annual temperature. The models testing the null expectation, that is, no relationship between species groups performed less well than the models testing for bottom‐up effects (results not shown). With increasing temperature, species richness in plant communities significantly increased, which in turn caused a significant increase in species richness of bird communities (Figure 2a + 3a, Table 1a). Similarly, mean annual temperature had a positive, indirect effect on bird abundance that was mediated by increased plant abundance (Figure 2b + 3b, Table 1b). Additionally, we found a weak, but significant direct relationship between mean annual temperature and species richness of birds, while mean annual precipitation explained no variation in plant or bird taxonomic diversity (Figure 2a + b).

**TABLE 1:** An overview of the model selection process and results for the structural equation models depicted in Figure 2: for (a) species richness, (b) species abundance, (c) functional richness (FRic), and (d) functional dispersion (FDis)

	Birds			Plants		Covariance	Model Fit	
	Plant	Tmean	Pmean	Tmean	Pmean	Bird–Plant	AICc	ΔAICc
(a) Species richness								
1	0.83***	0.18***	0.05	0.56**	0.02	—	100.78	6.22
2	0.83***	0.18***	0.05	0.56***	—	—	97.13	2.57
3	0.84***	0.16***	—	0.55***	—	—	94.56	0
(b) Species abundance								
1	0.99***	0.02	0.05	0.51**	0.18	—	89.66	8.11
2	0.99***	—	0.03	0.51**	0.18	—	86.37	4.82
3	0.99***	—	0.03	0.42**	—	—	83.76	2.21
4	0.99***	—	—	0.42**	—	—	81.55	0
(c) Functional richness								
1	0.17	0.47**	−0.09	0.21	0.59***	—	185.13	5.25
2	0.12	0.51***	—	0.21	0.59***	—	181.58	1.7
3	—	0.50**	—	0.21	0.59***	0.14	181.39	1.51
4	—	0.48**	—	—	0.48**	0.15	179.88	0
(d) Functional dispersion								
1	0.21	−0.44**	−0.71**	0.09	0.74***	—	169.67	3.15
2	0.21	−0.44**	−0.71**	—	0.68***	—	166.52	0
3	—	−0.44**	−0.57**	—	0.68***	0.16	166.52	0

Note

A stepwise removal of nonsignificant relationships led to a gradually better fit of the models. Error covariance between bird and plant communities was not explicitly assumed in the best‐fit model for species richness and species abundance (Table 1a + b). We tested this relationship separately and model fit decreased considerably. Given are the respective estimates for all possible paths in the models (see Figure 1a) as well as the AICc score and the distance of each model to the best‐fit model for each respective diversity metric. Significance levels of estimates: *p < 0.05, **p < 0.01; ***p < 0.001. Best‐fit models had a good fit to the data (in all cases: *P*(*χ* ^2^) > 0.05; lower 90% of confidence intervals of RMSEA close to 0; CFI > 0.95). *R* ^2^ values of the best‐fit models are shown in the respective figures (see Figure 2a–d).

Functional diversity measures of bird communities showed consistent, direct climatic effects and no significant relationships with the functional diversity measures of plant communities. Models performed better when the error covariance between species groups was included, but covariance terms were not significant for neither FRic nor FDis. Functional richness (FRic) of bird communities increased significantly with mean annual temperature, while FRic of plant communities was strongly positively linked to mean annual precipitation (Figure 2c + 3c,d, Table 1c). For both communities, the directions of the relationships were reversed when we calculated the standardized effect size (FRic_SES_), but these links were not statistically significant (see Appendix S2, Figure S2.3a + Table S2.1a). FDis of bird communities was negatively related to both mean annual temperature and precipitation, while increasing FDis of plant communities was positively related to mean annual precipitation (Figure 2d + 3e,f, Table 1d). These patterns of the raw FDis values were consistent with those for the standardized effect sizes (FDis_SES_, see Appendix S2, Figure S2.3b + Table S2.1b).

## 4 DISCUSSION

We compared patterns of taxonomic and functional diversity of frugivorous birds across two major mountain systems of the Neotropics and Afrotropics and simultaneously disentangled direct and indirect effects of climate and resource diversity. The overall diversity of fleshy‐fruited plants and avian frugivores was higher in the Ecuadorian Andes than on Mt. Kilimanjaro, which is in line with other studies showing similar differences between the two biogeographic regions (e.g., Hawkins et al., 2007; Kissling et al., 2012). Such patterns could be explained by different historical backgrounds or by distinct biogeographical properties. For instance, mountains with humid bases such as the Andes usually show different diversity patterns than mountains with arid bases such as Mt. Kilimanjaro (McCain, 2009). Despite these biogeographic differences, the mechanisms behind the diversity of fruits and frugivores across elevational gradients were very similar in both regions.

We show that in both biogeographic regions, the taxonomic diversity of birds was most strongly related to the taxonomic diversity of plants, while the functional diversity of birds was related to mean annual temperature and precipitation. Interestingly, the taxonomic diversity of plants was positively related to mean annual temperature and the functional diversity of plants was closely related to mean annual precipitation. Against our expectations, we did not find a link between the functional diversity of birds and plants, even though functional diversity was quantified based on matching traits that mediate bird‐plant interactions. These findings suggest that bottom‐up mechanisms can drive taxonomic diversity of avian frugivores across large environmental gradients, underlining the importance of resource tracking by frugivores. Trait matching between birds and their plant resources however did not result in spatial covariation in the functional diversity of plants and birds.

### 4.1 Bottom‐up mechanisms

Both species richness and abundance of bird communities were consistently linked to the corresponding measures of taxonomic diversity of their resources across large environmental gradients in the Neotropics and Afrotropics. These findings confirm our first hypothesis and are in line with previous findings (Ferger et al., 2014; Kissling et al., 2007; Loiselle & Blake, 1993). Our results underline how resources may affect the richness and abundance of frugivorous birds through bottom‐up mechanisms. The strong link between resource diversity and frugivore diversity at the taxonomic level hints at the particular importance of frugivore behavior, as active tracking is required in order to follow patterns of spatiotemporal fluctuations in the richness and abundance of fruit resources (Herrera, 1984, 1985).

Against our expectations, we did not detect relationships between the functional diversity of frugivore communities and their resources. In an earlier study from the Peruvian Andes, the functional diversity of fleshy‐fruited plants and frugivorous birds correlated closely across an elevational gradient covering natural habitat types from the lowlands to the tree line (Dehling et al., 2014). This pattern led the authors to propose that trait matching between resources and consumers caused bottom‐up effects of plants on avian frugivores. Trait matching between resources and avian frugivores has also been described for communities on Mt. Kilimanjaro (Vollstädt et al., 2017). In contrast to the here presented analysis, this study included human‐modified habitats, where a strong reduction of plant functional diversity caused decreasing functional diversity of frugivorous birds. A high functional diversity of frugivore communities could only be maintained if the functional diversity of resources was sufficiently high (Vollstädt et al., 2017).

Interestingly, we could not find similar dependencies of plant and bird functional diversity in near‐natural habitats across the two mountain systems in the Neotropics and the Afrotropics. Instead, direct effects of climate were relatively more important in shaping consumer diversity than biotic resource effects mediated by trait matching. Although the patterns of bird functional diversity were overall similar in both biogeographic regions, the functional diversity of plant communities was differently distributed across elevation. This difference is likely due to the different distribution of precipitation across the two elevational gradients, which is known to result in different patterns of diversity (e.g., McCain, 2009). Divergent responses of plant functional diversity in the Neotropics and Afrotropics might explain the relatively low explanatory power of our models for functional diversity (cf. R2 values in Figure 2), and why we could not detect effects of ecological fitting between resource and consumer species at the intercontinental scale.

### 4.2 Effects of climate

Climate explained much of the variation in functional diversity of both plant and bird communities. These findings are in contrast with our expectations and a previous study (Hanz et al., 2019), since our hypothesis was a higher importance of indirect effects through bottom‐up mechanisms and plant communities for diversity patterns of bird communities. Instead, we found that different climatic drivers were related to functional diversity of birds and plants. Whereas bird communities increased their functional trait space (but were less functionally dispersed) with increasing temperature, plant communities increased their functional trait space and were more functionally dispersed with higher precipitation.

It is well documented that water availability can drive the species richness of plants (Francis & Currie, 2003; Kreft & Jetz, 2007). The positive relationship between functional richness and precipitation suggests that the corresponding trait space of plant communities may expand under favorable climatic conditions. The increase in functional dispersion with increasing precipitation additionally suggests that the abundance of functionally distinct plant species increases in these communities, leading to functionally over‐dispersed plant communities with a potentially higher degree of niche partitioning. Commonly, patterns of over‐dispersion are attributed to competition among species, which results in the exclusion of functionally similar species and increasing functional specialization (Graham et al., 2009; Machac et al., 2011). We here included only traits that are directly related to seed dispersal by frugivores, such as fruit size and crop mass (Dehling et al., 2014; Wheelwright, 1985). The patterns we described for plant communities may thus primarily reflect competition for animal seed dispersers. This finding would be in line with previous work, where it has been shown that plants may compete for animal seed dispersers, especially when plant niche space becomes saturated (Albrecht et al., 2015).

In contrast, plant communities under harsh environmental conditions (i.e., low precipitation) showed signs of functional clustering, which is typically explained by environmental filtering (Machac et al., 2011; Pavoine et al., 2010; Webb, 2000). Plants under such conditions may possibly not be able to invest into attracting specific seed dispersers. Hence, there seems to be an important difference in the patterns of functional dispersion between plots with high and low precipitation, possibly due to differences in the degree of competition for seed dispersers. Although we find this pattern to be consistent on both mountain ranges, there are differences between the Ecuadorian Andes and Mt. Kilimanjaro. Functional dispersion of plant communities was overall lower in the Ecuadorian Andes than on Mt. Kilimanjaro (see Figure 3f). This pattern may be explained by a potentially stronger competition for high‐quality seed dispersers across the entire elevational range of Mt. Kilimanjaro, as there are comparatively fewer and less specialized avian frugivores in the Afrotropics than in the Neotropics (Dugger et al., 2019; Kissling et al., 2012), leading to overall higher levels of functional dispersion there. Interestingly, functional dispersion of bird communities was negatively related to precipitation. Functional dispersion of bird communities was overall lower while simultaneously precipitation was overall considerably higher in the Ecuadorian Andes than on Mt. Kilimanjaro (see Table S3.2). Previous work suggests that in the Ecuadorian Andes, bird diversity and abundance at low elevations is constrained by high precipitation (Santillán et al., 2018). Therefore, the negative relationship between the functional dispersion of bird communities and precipitation may arise, because only very competitive species with particular traits might be able to coexist locally under extreme rainfall.

Diversity and abundance of resources, as well as taxonomic and functional diversity of bird communities, were strongly and positively related to mean annual temperature. This pattern may be expected, since it is well established that temperature is a main driver of biodiversity worldwide (Evans et al., 2005), especially along tropical elevational gradients (Peters et al., 2016). Functional richness of bird communities was highest on plots characterized by warm temperatures and declined with decreasing temperatures across elevation. Similar patterns have been described for bird communities in the Peruvian Andes (Dehling et al., 2014; Pigot et al., 2016). Our results thus suggest that the pattern of increasing functional richness and, consequently, expansion of the functional trait space with increasing temperature may be generally true for Neotropical and Afrotropical mountains. However, under warm conditions, the size of bird functional trait spaces was smaller than expected from null models. This pattern was corroborated by the patterns of functional dispersion, which generally decreased with ambient temperature. Increasing temperatures, thus, promoted the assembly of communities that were composed mainly of functionally similar species, suggesting functional clustering. We hence find a pattern that is contrasting earlier work, since communities are typically described as functionally clustered under harsh environmental conditions (Dehling et al., 2014; Machac et al., 2011; Pigot et al., 2016). We, in contrast, demonstrate that communities on plots with lowest temperatures showed signs of functional over‐dispersion. This pattern may imply that frugivores might have to compete for highly limited resources in such harsh environmental conditions, leading to niche partitioning and functional specialization. Similar patterns were described in an earlier study on Mt. Kilimanjaro, where plant communities on warmer plots supported large communities of functionally similar frugivores, which overlapped in their resource use, while bird communities on colder plots apparently competed more strongly for resources (Vollstädt et al., 2017, 2018).

## 5 CONCLUSIONS

In the first simultaneous comparison of the effects of climate and resources on the diversity of avian frugivores between the Neotropics and Afrotropics, we show that bottom‐up mechanisms drive species richness and abundance of bird communities, but we do not find evidence for ecological fitting in terms of functional diversity. Functional diversity in communities of fleshy‐fruited plants and avian frugivores was related to different climatic drivers, showing distinct patterns of functional clustering and over‐dispersion across environmental gradients. The different patterns of functional over‐dispersion between fleshy‐fruited plants and frugivorous birds are remarkable, as they suggest distinct responses of resources and consumers to different climatic drivers. Consequently, ecological fitting between the functional traits of resource and consumer species may be masked at large spatial scales by decoupling of plant and bird functional diversity. Different responses of plant and bird functional diversity to climatic drivers could potentially further increase such decoupling between plants and animals under future conditions.

## CONFLICT OF INTEREST

None declared.

## AUTHOR CONTRIBUTIONS

**Maximilian G. R. Vollstädt:** Conceptualization (lead), data curation (lead), formal analysis (lead), funding acquisition (equal), investigation (lead), methodology (lead), project administration (lead), resources (lead), software (lead), supervision (lead), validation (lead), visualization (lead), writing – original draft (lead), writing – review and editing (lead). **Jörg Albrecht:** Conceptualization (supporting), data curation (supporting), formal analysis (equal), funding acquisition (supporting), investigation (supporting), methodology (equal), project administration (supporting), resources (supporting), software (supporting), supervision (supporting), validation (equal), visualization (equal), writing – original draft (equal), writing – review and editing (equal). **Katrin Böhning‐Gaese:** Conceptualization (equal), data curation (supporting), formal analysis (supporting), funding acquisition (lead), investigation (supporting), methodology (supporting), project administration (lead), resources (equal), software (equal), supervision (equal), validation (supporting), visualization (supporting), writing – original draft (supporting), writing – review and editing (supporting). **Andreas Hemp:** Conceptualization (supporting), data curation (supporting), formal analysis (supporting), funding acquisition (supporting), investigation (supporting), methodology (supporting), project administration (supporting), resources (supporting), software (supporting), supervision (supporting), validation (supporting), visualization (supporting), writing – original draft (supporting), writing – review and editing (supporting). **Kim M. Howell:** Conceptualization (supporting), data curation (supporting), formal analysis (supporting), funding acquisition (supporting), investigation (supporting), methodology (supporting), project administration (supporting), resources (supporting), software (supporting), supervision (supporting), validation (supporting), visualization (supporting), writing – original draft (supporting), writing – review and editing (supporting). **Laura Kettering:** Conceptualization (supporting), data curation (supporting), formal analysis (supporting), funding acquisition (supporting), investigation (supporting), methodology (supporting), project administration (lead), resources (supporting), software (supporting), supervision (supporting), validation (supporting), visualization (supporting), writing – original draft (supporting), writing – review and editing (supporting). **Alexander Neu:** Conceptualization (supporting), data curation (supporting), formal analysis (supporting), funding acquisition (supporting), investigation (supporting), methodology (supporting), project administration (supporting), resources (supporting), software (supporting), supervision (supporting), validation (supporting), visualization (supporting), writing – original draft (supporting), writing – review and editing (supporting). **Eike Lena Neuschulz:** Conceptualization (equal), data curation (equal), formal analysis (equal), funding acquisition (equal), investigation (equal), methodology (equal), project administration (equal), resources (equal), software (equal), supervision (equal), validation (equal), visualization (equal), writing – original draft (equal), writing – review and editing (equal). **Marta Quitián:** Conceptualization (supporting), data curation (supporting), formal analysis (supporting), funding acquisition (supporting), investigation (supporting), methodology (supporting), project administration (supporting), resources (supporting), software (supporting), supervision (supporting), validation (supporting), visualization (supporting), writing – original draft (supporting), writing – review and editing (supporting). **Vinicio E. Santillán:** Conceptualization (supporting), data curation (supporting), formal analysis (supporting), funding acquisition (supporting), investigation (supporting), methodology (supporting), project administration (supporting), resources (supporting), software (supporting), supervision (supporting), validation (supporting), visualization (supporting), writing – original draft (supporting), writing – review and editing (supporting). **Till Töpfer:** Conceptualization (supporting), data curation (supporting), formal analysis (supporting), funding acquisition (supporting), investigation (supporting), methodology (supporting), project administration (supporting), resources (supporting), software (supporting), supervision (supporting), validation (supporting), visualization (supporting), writing – original draft (supporting), writing – review and editing (supporting). **Matthias Schleuning:** Conceptualization (lead), data curation (equal), formal analysis (equal), funding acquisition (equal), investigation (equal), methodology (equal), project administration (equal), resources (equal), software (equal), supervision (equal), validation (equal), visualization (equal), writing – original draft (equal), writing – review and editing (equal). **Susanne A. Fritz:** Conceptualization (lead), data curation (lead), formal analysis (lead), funding acquisition (lead), investigation (lead), methodology (lead), project administration (lead), resources (lead), software (lead), supervision (lead), validation (lead), visualization (lead), writing – original draft (lead), writing – review and editing (lead).

## Supporting information

Figure S1a

Figure S1b

Figure S2

Appendix S1

Appendix S2

Appendix S3

## Data Availability

All data are available upon request from the corresponding author. Metadata of all Kilimanjaro datasets are also available online: https://www.kilimanjaro.biozentrum.uni‐wuerzburg.de/Data/Data.aspx. Metadata of all datasets of the Ecuadorian Andes are also available online: www.tropicalmountainforest.org.
