# Supplementary figures and images for: Direct and plant‐mediated effects of climate on bird diversity in tropical mountains
Source: Ecol Evol. 2020 Nov 13;10(24):14196–208. doi: 10.1002/ece3.7014 (PMC7771156; doi:10.1002/ece3.7014)

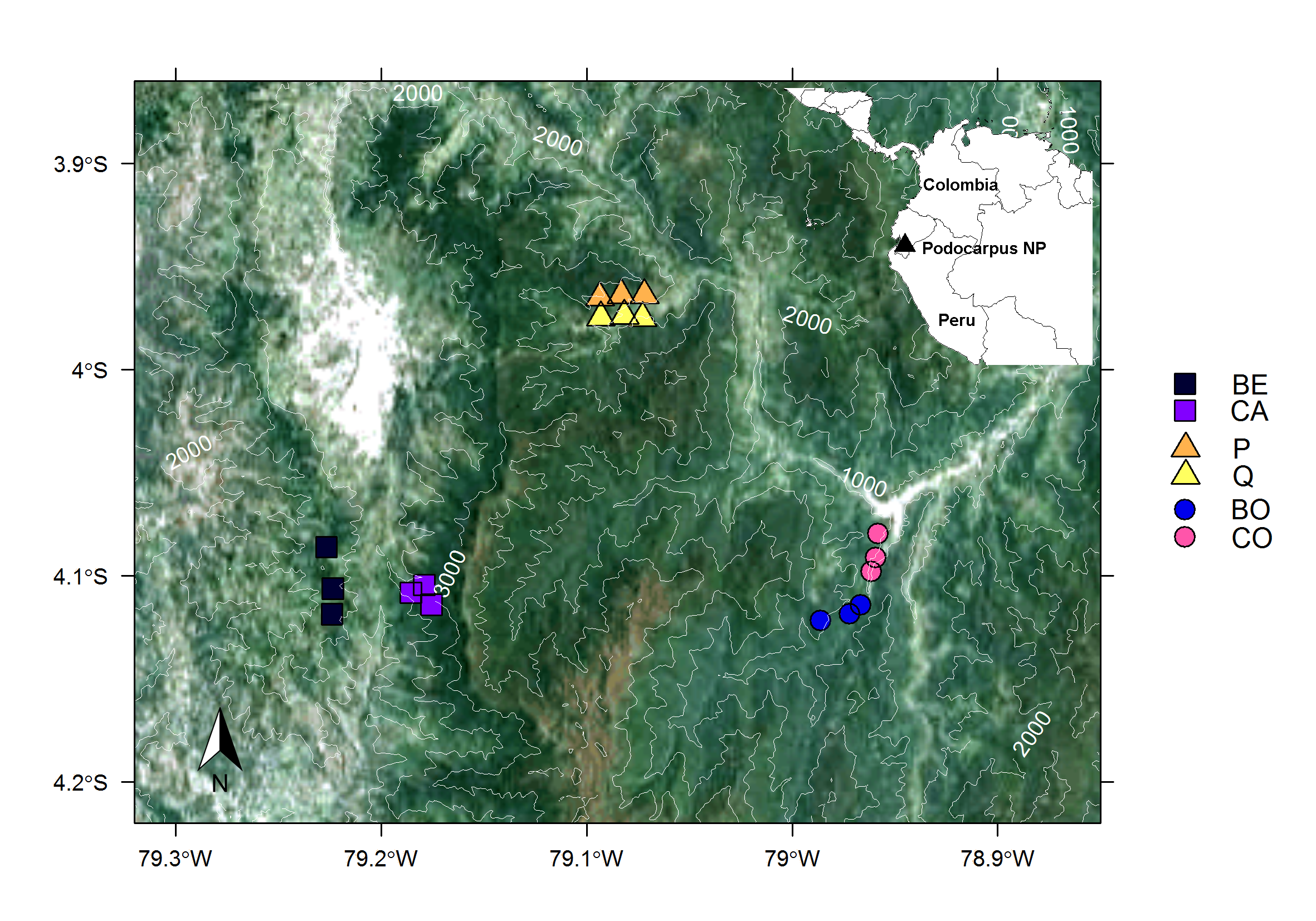

Supplement: Supplementary file 1 — Figure S1a [file ECE3-10-14196-s001.png]

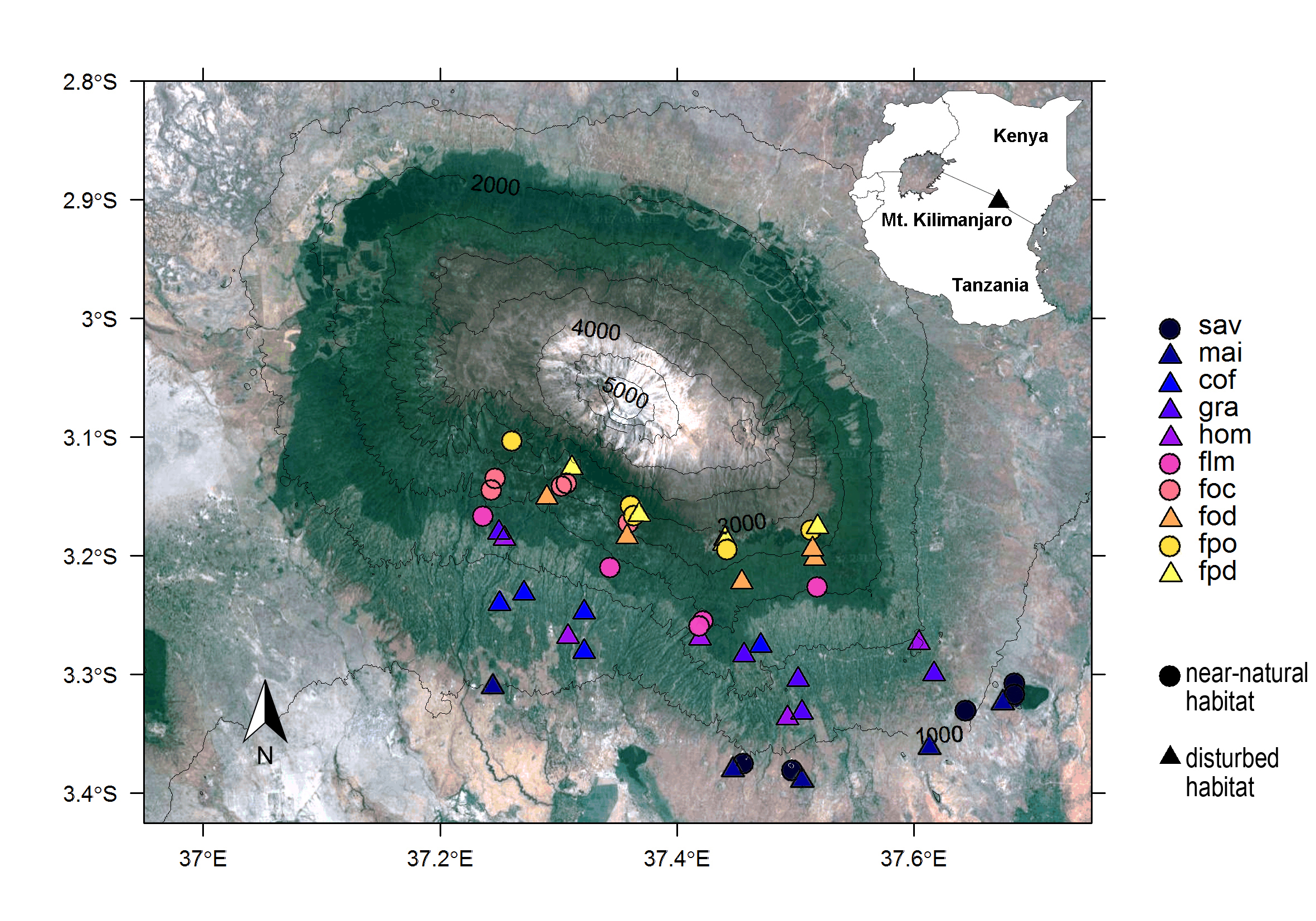

Supplement: Supplementary file 2 — Figure S1b [file ECE3-10-14196-s002.png]

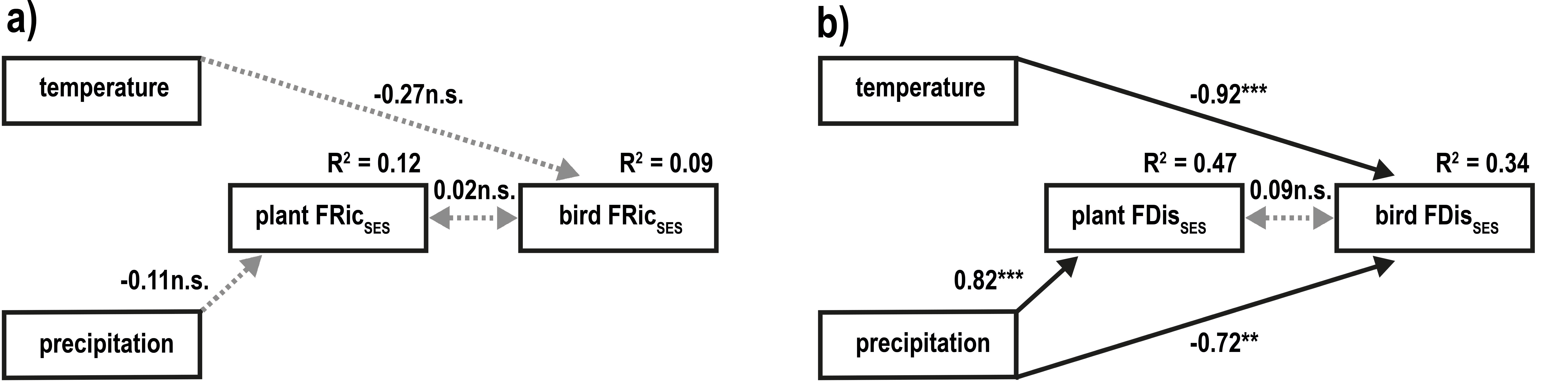

Supplement: Supplementary file 3 — Figure S2 [file ECE3-10-14196-s003.png]
