# Supplementary material for: Direct and plant‐mediated effects of climate on bird diversity in tropical mountains
Source: Ecol Evol. 2020 Nov 13;10(24):14196–208. doi: 10.1002/ece3.7014 (PMC7771156; doi:10.1002/ece3.7014)
Supplement: Supplementary file 4 — Appendix S1 [file ECE3-10-14196-s004.docx]

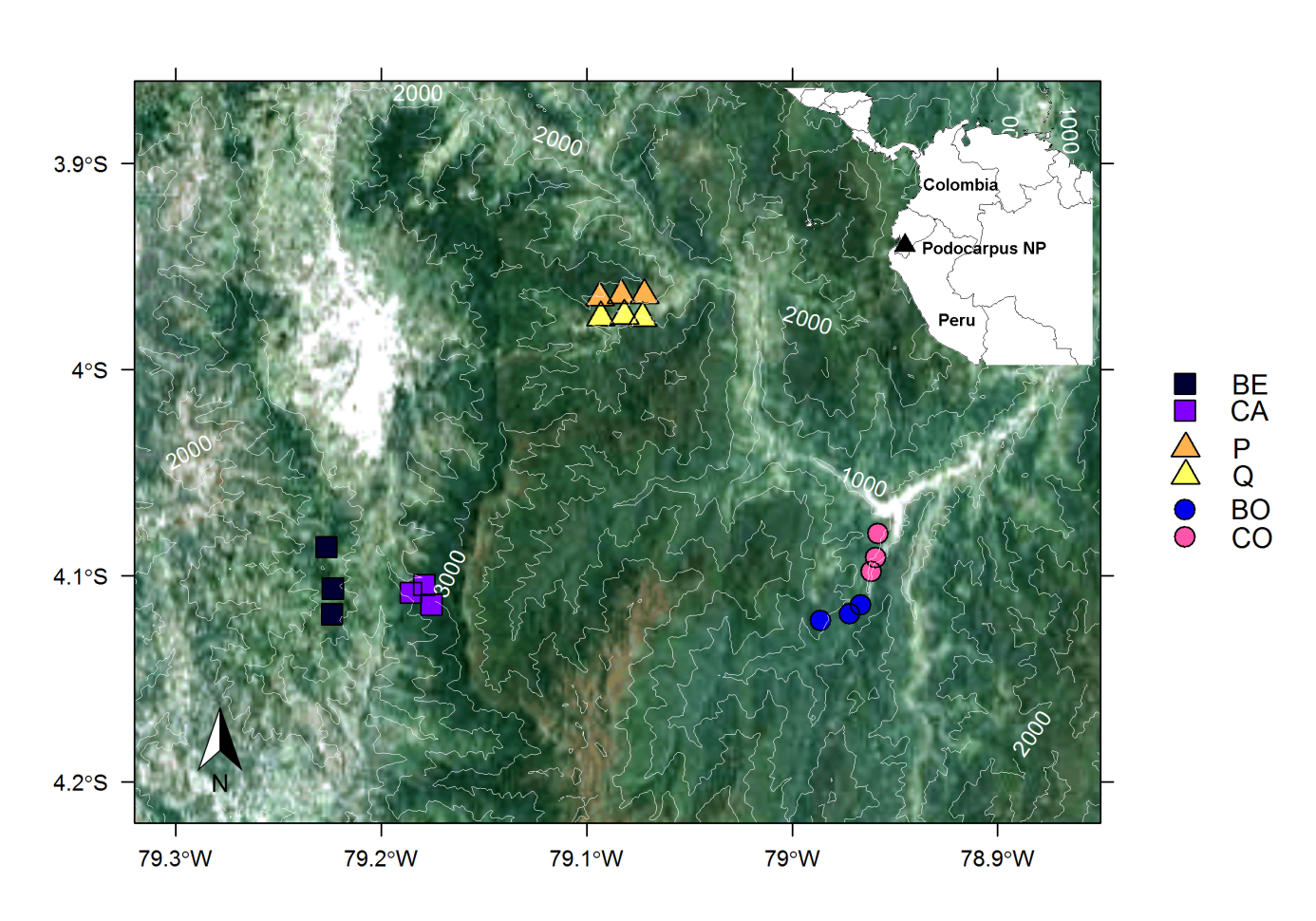


Figure S1.1: Map of the study area in the Ecuadorian Andes, showing the location of the 18 study plots. The different vegetation types are represented by different symbols. Circles: evergreen premontane forest (BO, CO, 1000 m a.s.l.), triangles: evergreen lower montane forest (Q, P, 2000 m a.s.l.) and boxes: upper montane forest (CA, BE, 3000 m a.s.l.). Map Data: Google TerraMetrics. Available online: http://maps.googleapis.com/maps/api/staticmap?center=-4,-79.1&zoom=10&size=640x497&maptype=satellite&format=gif&sensor=false&scale=2 (accessed on 16 September 2017). Digital elevation model: <https://lta.cr.usgs.gov/SRTM1Arc>.


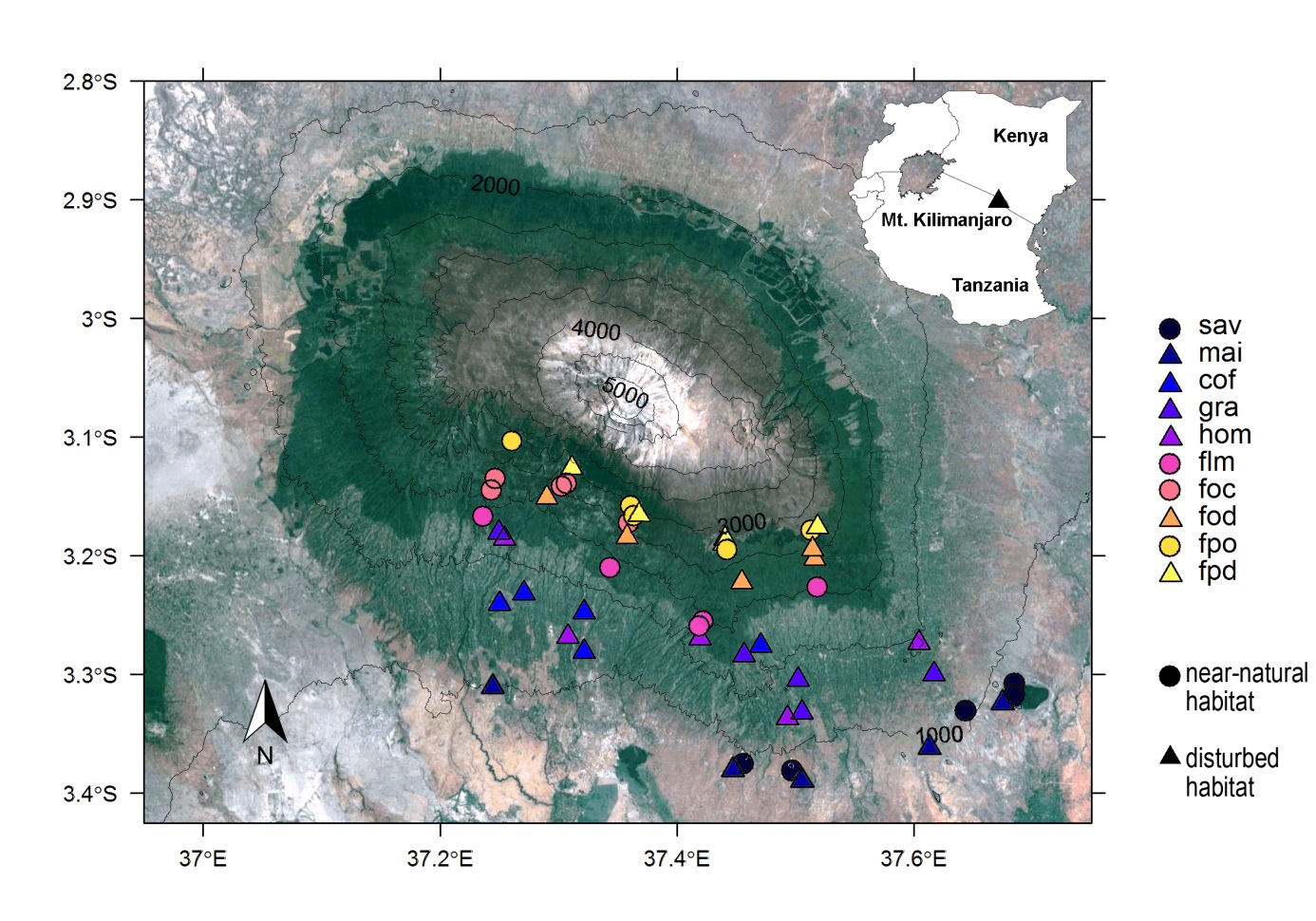


Figures S1.2: Map of Mt. Kilimanjaro, showing the location of 50 study plots. For this study only near-natural habitat types were included in the analysis. Plots from near-natural habitat types are represented by circles: savanna (sav), lower montane forest (flm), *Ocotea* forest (foc) and *Podocarpus* forest (fpo). Map Data: Google TerraMetrics. Available online: http://maps.googleapis.com/maps/api/staticmap?center=-3.123553240247,37.366348380164&zoom=10&size=640x497&maptype=satellite&format=gif&sensor=false&scale=2 (accessed on 24 August 2017). Digital elevation model: <https://lta.cr.usgs.gov/SRTM1Arc>.
