# Supplementary material for: Direct and plant‐mediated effects of climate on bird diversity in tropical mountains
Source: Ecol Evol. 2020 Nov 13;10(24):14196–208. doi: 10.1002/ece3.7014 (PMC7771156; doi:10.1002/ece3.7014)
Supplement: Supplementary file 5 — Appendix S2 [file ECE3-10-14196-s005.docx]

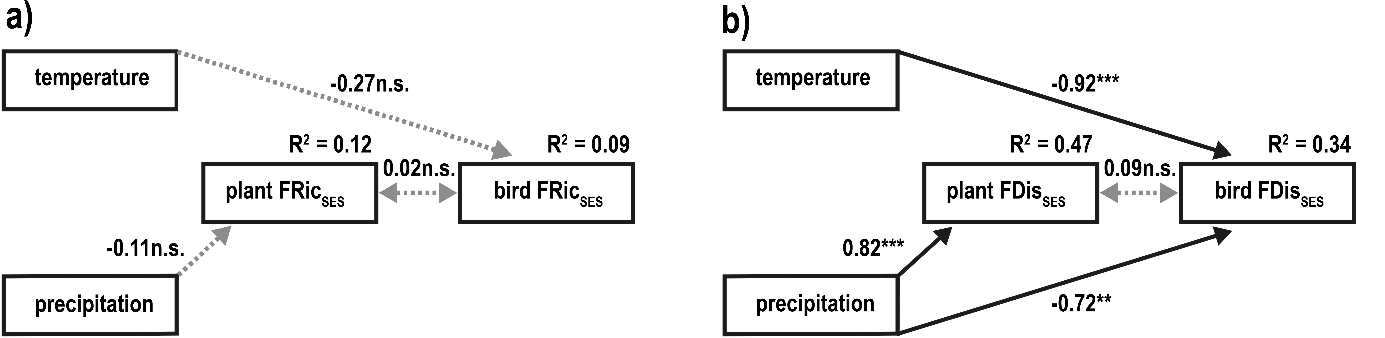


Figure S2.3: Relationships between bird communities, plant resource communities and climate (temperature and precipitation) in the Ecuadorian Andes and on Mt. Kilimanjaro, according to the models with highest model fit for a) effect size of functional richness (FRic_SES_) and b) effect size of functional dispersion (FDis_SES_). The path coefficients for paths in the best fit model, their statistical significance (**P* < 0.05, ***P* < 0.01, ****P* < 0.001) and the coefficients of determination (*R*^2^) are given. Non-significant paths that were retained in the best models are represented by dotted lines.

Table S2.1: An overview of the model selection process and results for the structural equation models depicted in Figure S2.3: for a) effect size of functional richness (FRic_SES_) and b) effect size of functional dispersion (FDis_SES_). A stepwise removal of non-significant relationships led to a gradually better fit of the models. Given are the respective estimates for all possible paths in the models (see Fig. 1c) as well as the AICc score and the distance of each model to the best-fit model for each respective diversity metric. Significance levels of estimates: *P < 0.05, **P < 0.01; ***P < 0.001. Best-fit models had a good fit to the data (in all cases: P(χ^2^) > 0.05; lower 90% of confidence intervals of RMSEA close to 0; CFI > 0.95).

|  | Birds | | | Plants | | Covariance | Model Fit | |  |
| --- | --- | --- | --- | --- | --- | --- | --- | --- | --- |
|  | Plant | Tmean | Pmean | Tmean | Pmean | Bird - Plant | AICc | ΔAICc | |
| a) Functional richness effect size | | | | |  |  |  |  | |
| 1 | 0.11 | **-0.39*** | -0.23 | -0.02 | -0.13 | - | 149.52 | 5.68 | |
| 2 | 0.11 | **-0.39*** | -0.23 | - | -0.12 | - | 145.83 | 1.99 | |
| 3 | - | **-0.39*** | -0.25 | - | -0.12 | 0.02 | 145.83 | 1.99 | |
| 4 | - | -0.27 | - | - | -0.11 | 0.02 | 143.84 | 0 | |
| b) Functional dispersion effect size | | | | |  |  |  |  | |
| 1 | 0.15 | **-0.92***** | **-0.84**** | 0.13 | **0.90***** | - | 208.39 | 2.94 | |
| 2 | - | **-0.89***** | **-0.71**** | 0.13 | **0.90***** | - | 208.39 | 2.94 | |
| 3 | - | **-0.92***** | **-0.72**** | - | **0.82***** | 0.09 | 205.45 | 0 | |
