# Supplementary material for: Direct and plant‐mediated effects of climate on bird diversity in tropical mountains
Source: Ecol Evol. 2020 Nov 13;10(24):14196–208. doi: 10.1002/ece3.7014 (PMC7771156; doi:10.1002/ece3.7014)
Supplement: Supplementary file 6 — Appendix S3 [file ECE3-10-14196-s006.docx]

Table S3.2: List of all variables included in the structural equation models for the Ecuadorian Andes and Mt. Kilimanjaro. Given are means, standard deviation as well as minimum and maximum values for each variable. Temperature and precipitation are mean annual values. As measures of taxonomic diversity, we used species richness (Spec. ric.) and species abundance (Spec. abun.) As multiple-trait indices of functional diversity, we used functional richness (FRic) and functional dispersion (FDis).

|  | Mean | SD | Minimum | Maximum |
| --- | --- | --- | --- | --- |
| Ecuadorian Andes |  |  |  |  |
| Temperature [° C] | 15.58 | 3.82 | 10.28 | 20.35 |
| Precipitation [mm] | 2331.94 | 610.65 | 1704 | 3479 |
| Elevation [m asl] | 1898.24 | 738.59 | 1000 | 3000 |
| Spec. ric. plants | 13.47 | 3.36 | 8 | 22 |
| Spec. ric. birds | 24 | 14.89 | 2 | 53 |
| Spec. abun. plants | 413203.2 | 352842.8 | 4730 | 1150068 |
| Spec. abun. birds | 321.29 | 365.01 | 8 | 1282 |
| FRic plants | 0.025 | 0.019 | 0.003 | 0.076 |
| FRic birds | 0.068 | 0.063 | 0.000 | 0.202 |
| FDis plants | 1.454 | 0.177 | 1.120 | 1.176 |
| FDis birds | 1.375 | 0.305 | 0.814 | 2.027 |
| Mt. Kilimanjaro |  |  |  |  |
| Temperature [° C] | 15.91 | 4.06 | 9.5 | 21.5 |
| Precipitation [mm] | 1653.17 | 625.19 | 609 | 2653 |
| Elevation [m asl] | 1917.72 | 714.41 | 871 | 2833 |
| Spec. ric. plants | 8.28 | 2.85 | 5 | 15 |
| Spec. ric. birds | 13.17 | 4.29 | 7 | 23 |
| Spec. abun. plants | 266400.7 | 507774.9 | 1690 | 1686335 |
| Spec. abun. birds | 244.05 | 146.86 | 68 | 585 |
| FRic plants | 0.011 | 0.155 | <0.001 | 0.063 |
| FRic birds | 0.049 | 0.051 | 0.003 | 0.202 |
| FDis plants | 1.869 | 0.454 | 0.717 | 2.382 |
| FDis birds | 1.624 | 0.247 | 2.064 | 1.269 |

Table S3.3: List of fleshy-fruited plant species recorded on all study plots of the Ecuadorian Andes and Mt. Kilimanjaro. Plants were recorded between November 2013 and October 2015. Given is the number of individuals of the respective species that frugivorous birds interacted with, pooled over all plots. Range gives the lowest elevation and the highest elevation a plant species was observed. Plants are ordered alphabetically within the respective taxonomic division.

| Scientific Name | Family | N Interactions | Range [m asl] |
| --- | --- | --- | --- |
| Ecuadorian Andes |  |  |  |
| Magnoliopsida |  |  |  |
| *Viburnum obtectum* | Adoxaceae | 58 | 2000 |
| *Guatteria sp1* | Annonaceae | 1 | 1000 |
| *Oreopanax sp 1* | Araliaceae | 5 | 3000 |
| *Schefflera sp 1* | Araliaceae | 185 | 1000 |
| *Schefflera sp 2* | Araliaceae | 1 | 2000 |
| *Schefflera sp 3* | Araliaceae | 10 | 3000 |
| *Schefflera sp 4* | Araliaceae | 1 | 3000 |
| *Schefflera acuminata* | Araliaceae | 9 | 3000 |
| *Tournefortia sp 1* | Boraginaceae | 1 | 3000 |
| *Dacryodes sp 1* | Bursercaceae | 12 | 1000 |
| *Trema micrantha* | Cannabaceae | 1 | 2000 |
| *Hedyosmum sp 1* | Chloranthaceae | 2 | 3000 |
| *Ericaceae sp 1* | Ericaceae | 10 | 3000 |
| *Ericaceae sp 2* | Ericaceae | 4 | 3000 |
| *Ericaceae sp 3* | Ericaceae | 1 | 3000 |
| *Ericaceae sp 4* | Ericaceae | 4 | 3000 |
| *Ericaceae sp 5* | Ericaceae | 3 | 3000 |
| *Gaultheria erecta* | Ericaceae | 1 | 3000 |
| *Escallonia paniculata* | Escalloniaceae | 2 | 2000 |
| *Alchornea sp 1* | Euphorbiaceae | 18 | 1000 |
| *Sapium cf glandulosum* | Euphorbiaceae | 6 | 2000 |
| *Inga sp 1* | Fabaceae | 3 | 1000 |
| *Inga sp 2* | Fabaceae | 3 | 1000 |
| *Inga sp 3* | Fabaceae | 1 | 2000 |
| *Ocotea sp 1* | Lauraceae | 2 | 3000 |
| *Persea weberbaueri* | Lauraceae | 2 | 2000 |
| *Loranthaceae sp 1* | Loranthaceae | 1 | 2000 |
| *Mollia sp 1* | Malvaceae | 12 | 3000 |
| *Brachyotum sp 1* | Melastomataceae | 1 | 2000 |
| *Graffenrieda emarginata* | Melastomataceae | 6 | 3000 |
| *Graffenrieda harlingii* | Melastomataceae | 2 | 3000 |
| *Meriania sp 1* | Melastomataceae | 1 | 1000 |
| *Miconia sp 1* | Melastomataceae | 59 | 1000 |
| *Miconia sp 2* | Melastomataceae | 41 | 1000 |
| *Miconia sp 3* | Melastomataceae | 6 | 1000 |
| *Miconia sp 4* | Melastomataceae | 2 | 1000 |
| *Miconia sp 5* | Melastomataceae | 52 | 1000 |
| *Miconia sp 6* | Melastomataceae | 4 | 1000 |
| *Miconia sp 7* | Melastomataceae | 779 | 1000 |
| *Miconia sp 8* | Melastomataceae | 13 | 1000 |
| *Miconia sp 9* | Melastomataceae | 191 | 1000 |
| *Miconia sp 10* | Melastomataceae | 384 | 1000 |
| *Miconia sp 11* | Melastomataceae | 2 | 1000 |
| *Miconia sp 12* | Melastomataceae | 17 | 1000 |
| *Miconia sp 13* | Melastomataceae | 762 | 1000 |
| *Miconia sp 14* | Melastomataceae | 1 | 1000 |
| *Miconia sp 15* | Melastomataceae | 3 | 2000 |
| *Miconia sp 16* | Melastomataceae | 6 | 2000 |
| *Miconia sp 17* | Melastomataceae | 1 | 2000 |
| *Miconia sp 18* | Melastomataceae | 4 | 3000 |
| *Miconia sp 19* | Melastomataceae | 8 | 3000 |
| *Miconia sp 20* | Melastomataceae | 6 | 3000 |
| *Miconia sp 21* | Melastomataceae | 3 | 3000 |
| *Miconia sp 22* | Melastomataceae | 7 | 3000 |
| *Miconia sp 23* | Melastomataceae | 32 | 3000 |
| *Miconia sp 24* | Melastomataceae | 1 | 3000 |
| *Miconia punctata* | Melastomataceae | 183 | 2000 |
| *Miconia radula* | Melastomataceae | 3 | 3000 |
| *Miconia theaezans* | Melastomataceae | 721 | 2000 |
| *Tibouchina oxypetala* | Melastomataceae | 14 | 1000 |
| *Cedrela nebulosa* | Meliaceae | 1 | 2000 |
| *Morella pubescens* | Myricaceae | 6 | 3000 |
| *Fuchsia lehmannii* | Onagraceae | 2 | 2000 |
| *Hieronyma cf macrocarpa* | Phyllanthaceae | 2 | 3000 |
| *Hieronyma fendleri* | Phyllanthaceae | 6 | 2000 |
| *Piper sp 1* | Piperaceae | 2 | 1000 |
| *Piper sp 2* | Piperaceae | 8 | 1000 |
| *Monnina sp1* | Polygalaceae | 6 | 2000 |
| *Myrsine coriacea* | Primulaceae | 674 | 1000-2000 |
| *Rubus sp 1* | Rosaceae | 24 | 2000 |
| *Rubus sp 2* | Rosaceae | 15 | 3000 |
| *Faramea sp 1* | Rubiaceae | 1 | 2000 |
| *Guettarda hirsuta* | Rubiaceae | 2 | 3000 |
| *Guettarda sp 1* | Rubiaceae | 4 | 1000 |
| *Isertia laevis* | Rubiaceae | 34 | 1000-2000 |
| *Nertera depressa* | Rubiaceae | 1 | 3000 |
| *Palicourea sp 1* | Rubiaceae | 1 | 2000 |
| *Palicourea sp 2* | Rubiaceae | 7 | 2000 |
| *Palicourea sp 3* | Rubiaceae | 1 | 3000 |
| *Palicourea sp 4* | Rubiaceae | 1 | 3000 |
| *Palicourea thyrsiflora* | Rubiaceae | 4 | 2000 |
| *Rubiaceae sp 1* | Rubiaceae |  | 1000 |
| *Allophylus sp 1* | Sapindaceae | 3 | 2000 |
| *Allophylus sp 2* | Sapindaceae | 3 | 3000 |
| *Siparuna sp 1* | Siparunaceae | 1 | 1000 |
| *Siparuna aspera* | Siparunaceae | 9 | 2000 |
| *Turpinia occidentalis* | Staphyleaceae | 5 | 2000 |
| *Symplocos sulcinervius* | Symplocaceae | 1 | 3000 |
| *Gordonia sp 1* | Theaceae | 1 | 3000 |
| *Cecropia sp 1* | Urticaceae | 2 | 1000 |
| *Cecropia sp 2* | Urticaceae | 492 | 1000 |
| *Cecropia sp 3* | Urticaceae | 8 | 1000 |
| *Cecropia andina* | Urticaceae | 1 | 2000 |
| *Cecropia angustifolia* | Urticaceae | 144 | 2000 |
| *Myriocarpa sp 1* | Urticaceae | 1 | 3000 |
| *Urera sp 1* | Urticaceae | 6 | 1000 |
| *Indet* |  |  |  |
| *Indet 1* | Indet | 1 | 1000 |
| *Indet 2* | Indet | 1 | 1000 |
| *Indet 3* | Indet | 117 | 1000 |
| *Indet 4* | Indet | 18 | 1000 |
| *Indet 5* | Indet | 3 | 1000 |
| *Indet 6* | Indet | 100 | 1000 |
| *Indet 7* | Indet | 1 | 2000 |
| *Indet 8* | Indet | 3 | 2000 |
| *Indet 9* | Indet | 4 | 3000 |
| *Indet 10* | Indet | 2 | 3000 |
| *Indet 11* | Indet | 1 | 3000 |
| *Indet 12* | Indet | 3 | 2000 |
| *Indet 13* | Indet | 3 | 2000 |
| *Indet 14* | Indet | 1 | 3000 |
| *Indet 15* | Indet | 34 | 2000 |
| *Indet 16* | Indet | 2 | 1000 |
| *Indet 17* | Indet | 1 | 3000 |
| *Indet 18* | Indet | 1 | 2000 |
| Mt. Kilimanjaro |  |  |  |
| Pinopsida |  |  |  |
| *Podocarpus latifolius* | Podocarpaceae | 56 | 2370–2990 |
| Liliopsida |  |  |  |
| *Dracaena afromontana* | Asparagaceae | 1 | 1650 |
| *Smilax anceps* | Smilacaceae | 59 | 1300–1750 |
| *Magnoliopsida* |  |  |  |
| *Lannea schimperi* | Anacardiaceae | 307 | 871–1130 |
| *Ozoroa insignis* | Anacardiaceae | 91 | 871–993 |
| *Sclerocarya birrea* | Anacardiaceae | 2 | 993 |
| *Ilex mitis* | Aquifoliaceae | 24 | 2650–2940 |
| *Schefflera myriantha* | Araliaceae | 337 | 1920–2820 |
| *Schefflera volkensii* | Araliaceae | 2573 | 2040–2970 |
| *Begonia meyeri-johannis* | Begoniaceae | 16 | 1650–2540 |
| *Commiphora africana* | Burseraceae | 73 | 871–992 |
| *Commiphora sp.* | Burseraceae | 5 | 950 |
| *Commiphora triphylla* | Burseraceae | 35 | 992 |
| *Trema orientalis* | Cannabaceae | 434 | 1620–1920 |
| *Maytenus acuminata* | Celastraceae | 12 | 1920–2940 |
| *Peponium vogelei* | Cucurbitaceae | 4 | 1650 |
| *Zehneria scabra* | Cucurbitaceae | 65 | 2850–2940 |
| *Bridelia cathartica* | Euphorbiaceae | 12 | 871–912 |
| *Flueggea virosa* | Euphorbiaceae | 29 | 962–993 |
| *Macaranga capensis* | Euphorbiaceae | 210 | 1650–2120 |
| *Macaranga kilimandscharica* | Euphorbiaceae | 258 | 1650–2270 |
| *Ocotea usambarensis* | Lauraceae | 89 | 1620–2750 |
| *Emelianthe panganensis* | Loranthaceae | 19 | 871–912 |
| *Erianthemum dregei* | Loranthaceae | 160 | 871–2560 |
| *Helixanthera kirkii* | Loranthaceae | 66 | 912 |
| *Plicosepalus curviflorus* | Loranthaceae | 52 | 993–1300 |
| *Tiliacora funifera* | Menispermaceae | 68 | 1650–2120 |
| *Ficus natalensis* | Moraceae | 51 | 912 |
| *Ficus sur* | Moraceae | 98 | 1620–1650 |
| *Maesa lanceolata* | Myrsinaceae | 118 | 1620–1650 |
| *Embelia schimperi* | Primulaceae | 28 | 2370–2940 |
| *Myrsine africana* | Primulaceae | 33 | 2770–2940 |
| *Rhamnus staddo* | Rhamnaceae | 52 | 1130 |
| *Ziziphus mucronata* | Rhamnaceae | 13 | 950 |
| *Prunus africana* | Rosaceae | 210 | 2800–2940 |
| *Rubus steudneri* | Rosaceae | 50 | 1800 –2880 |
| *Galiniera saxifraga* | Rubiaceae | 80 | 1800–2650 |
| *Heinsenia diervilleoides* | Rubiaceae | 10 | 1650 |
| *Keetia gueinzii* | Rubiaceae | 402 | 1650–1920 |
| *Lasianthus kilimandscharicus* | Rubiaceae | 47 | 1800–2260 |
| *Pauridiantha paucinervis* | Rubiaceae | 247 | 1650–2120 |
| *Psychotria cyathicalyx* | Rubiaceae | 248 | 1800–2800 |
| *Psychotria fractinervata* | Rubiaceae | 58 | 1800–2560 |
| *Psychotria petiginosa* | Rubiaceae | 16 | 2120–2650 |
| *Rytigynia uhligii* | Rubiaceae | 5 | 1920 |
| *Harrisonia abyssinica* | Rutaceae | 9 | 1130 |
| *Allophyllus ferrugineus* | Sapindaceae | 4 | 2040 |
| *Discopodium penninervum* | Solanaceae | 34 | 2120–2990 |
| *Lantana camara* | Verbenaceae | 885 | 871–1750 |
| *Cissus oliveri* | Vitaceae | 18 | 1307–1800 |
| *Cissus quadrangularis* | Vitaceae | 24 | 950–1130 |
| *Cyphostemma serpens* | Vitaceae | 11 | 871 |
| *Cyphostemma sp.* | Vitaceae | 9 | 871 |

Table S3.4: List of the fruit-eating bird species observed on all study plots of the Ecuadorian Andes and Mt. Kilimanjaro. Birds were observed during the study period between November 2013 and October 2015. Given is the number of individuals that have been observed handling fruits, pooled over all study plots. The range gives the lowest elevation and the highest elevation a species has been observed at. Birds are listed in alphabetical order within the respective family.

| Scientific Name | Family | N interactions | | Range [m asl] |
| --- | --- | --- | --- | --- |
| Ecuadorian Andes |  | |  |  |
| *Chamaepetes goudotii* | Cracidae | | 6 | 1000-2000 |
| *Ortalis guttata* | Cracidae | | 12 | 1000 |
| *Penelope barbata* | Cracidae | | 13 | 2000-3000 |
| *Columba fasciata* | Columbidae | | 19 | 2000 |
| *Columba plumbea* | Columbidae | | 11 | 1000 |
| *Columba subvinacea* | Columbidae | | 3 | 1000 |
| *Pharomachrus auriceps* | Trogonidae | | 2 | 2000 |
| *Trogon collaris* | Trogonidae | | 5 | 1000 |
| *Trogon personatus* | Trogonidae | | 1 | 2000 |
| *Aulacorhynchus prasinus* | Ramphastidae | | 11 | 2000-3000 |
| *Eubucco bourcierii* | Ramphastidae | | 6 | 1000 |
| *Dryocopus lineatus* | Picidae | | 1 | 1000 |
| *Pionus menstruus* | Psittacidae | | 5 | 1000 |
| *Pyrrhura albipectus* | Psittacidae | | 114 | 1000 |
| *Lepidocolaptes lacrymiger* | Dendrocolaptidae | | 2 | 2000 |
| *Anairetes parulus* | Tyrannidae | | 1 | 2000 |
| *Elaenia albiceps* | Tyrannidae | | 35 | 2000 |
| *Elaenia pallatangae* | Tyrannidae | | 13 | 2000 |
| *Leptopogon superciliaris* | Tyrannidae | | 9 | 1000 |
| *Mionectes olivaceus* | Tyrannidae | | 25 | 1000-2000 |
| *Mionectes striaticollis* | Tyrannidae | | 2 | 1000-2000 |
| *Myiarchus cephalotes* | Tyrannidae | | 2 | 1000 |
| *Myiophobus pulcher* | Tyrannidae | | 3 | 2000 |
| *Myiozetetes granadensis* | Tyrannidae | | 1 | 1000 |
| *Myiozetetes similis* | Tyrannidae | | 5 | 1000 |
| *Pogonotriccus poecilotis* | Tyrannidae | | 1 | 1000 |
| *Sayornis nigricans* | Tyrannidae | | 3 | 1000 |
| *Tyrannus melancholicus* | Tyrannidae | | 3 | 1000 |
| *Tityra semifasciata* | Tyrannidae | | 3 | 1000 |
| *Ampelion rubrocristatus* | Cotingidae | | 9 | 3000 |
| *Pipreola arcuata* | Cotingidae | | 3 | 3000 |
| *Pipreola chlorolepidota* | Cotingidae | | 2 | 1000 |
| *Pipreola frontalis* | Cotingidae | | 10 | 1000 |
| *Pipreola rieferii* | Cotingidae | | 1 | 2000 |
| *Rupicola peruviana* | Cotingidae | | 23 | 1000-2000 |
| *Dixiphia pipra* | Pipridae | | 41 | 1000 |
| *Lepidothrix isidorei* | Pipridae | | 44 | 1000 |
| *Machaeropterus striolatus* | Pipridae | | 23 | 1000 |
| *Manacus manacus* | Pipridae | | 1 | 1000 |
| *Pipra erythrocephala* | Pipridae | | 68 | 1000 |
| *Cyclarhis gujanensis* | Vireonidae | | 2 | 2000 |
| *Hylophilus olivaceus* | Vireonidae | | 1 | 1000 |
| *Vireo leucophrys* | Vireonidae | | 1 | 2000 |
| *Cyanocorax yncas* | Corvidae | | 62 | 1000-2000 |
| *Cyanolyca turcosa* | Corvidae | | 1 | 3000 |
| *Catharus ustulatus* | Turdidae | | 97 | 1000-2000 |
| *Myadestes ralloides* | Turdidae | | 25 | 1000-2000 |
| *Platycichla leucops* | Turdidae | | 8 | 1000 |
| *Turdus albicollis* | Turdidae | | 35 | 1000 |
| *Turdus fulviventris* | Turdidae | | 9 | 1000-2000 |
| *Turdus fuscater* | Turdidae | | 64 | 2000-3000 |
| *Turdus ignobilis* | Turdidae | | 1 | 1000 |
| *Turdus nigriceps* | Turdidae | | 1 | 1000 |
| *Turdus serranus* | Turdidae | | 1 | 2000 |
| *Ammodramus aurifrons* | Emberizidae | | 2 | 1000 |
| *Arremon aurantiirostris* | Emberizidae | | 3 | 1000 |
| *Atlapetes latinuchus* | Emberizidae | | 17 | 2000-3000 |
| *Buarremon torquatus* | Emberizidae | | 3 | 3000 |
| *Catamenia inornata* | Emberizidae | | 6 | 2000 |
| *Zonotrichia capensis* | Emberizidae | | 24 | 3000 |
| *Cacicus uropygialis* | Icteridae | | 7 | 2000 |
| *Psarocolius angustifrons* | Icteridae | | 116 | 1000 |
| *Psarocolius decumanus* | Icteridae | | 64 | 1000 |
| *Basileuterus tristriatus* | Parulidae | | 2 | 1000 |
| *Myioborus melanocephalus* | Parulidae | | 1 | 2000 |
| *Myioborus miniatus* | Parulidae | | 1 | 3000 |
| *Parula pitiayumi* | Parulidae | | 17 | 2000 |
| *Saltator grossus* | Cardinalidae | | 1 | 1000 |
| *Saltator maximus* | Cardinalidae | | 20 | 1000 |
| *Anisognathus igniventris* | Thraupidae | | 10 | 1000 |
| *Anisognathus lacrymosus* | Thraupidae | | 60 | 3000 |
| *Anisognathus somptuosus* | Thraupidae | | 74 | 2000-3000 |
| *Buthraupis eximia* | Thraupidae | | 1 | 2000 |
| *Buthraupis montana* | Thraupidae | | 11 | 2000 |
| *Chlorochrysa calliparaea* | Thraupidae | | 42 | 3000 |
| *Chlorophanes spiza* | Thraupidae | | 53 | 1000 |
| *Chlorornis riefferii* | Thraupidae | | 12 | 1000 |
| *Chlorospingus canigularis* | Thraupidae | | 51 | 2000-3000 |
| *Chlorospingus flavigularis* | Thraupidae | | 397 | 1000-2000 |
| *Chlorospingus ophthalmicus* | Thraupidae | | 4 | 1000 |
| *Cissopis leveriana* | Thraupidae | | 9 | 2000 |
| *Coereba flaveola* | Thraupidae | | 13 | 1000 |
| *Conirostrum albifrons* | Thraupidae | | 1 | 1000 |
| *Creurgops verticalis* | Thraupidae | | 21 | 3000 |
| *Dacnis cayana* | Thraupidae | | 12 | 2000 |
| *Dacnis flaviventer* | Thraupidae | | 1 | 1000 |
| *Dacnis lineata* | Thraupidae | | 117 | 1000 |
| *Diglossa albilatera* | Thraupidae | | 25 | 1000 |
| *Diglossa humeralis* | Thraupidae | | 1 | 2000-3000 |
| *Diglossopis caerulescens* | Thraupidae | | 1 | 3000 |
| *Diglossopis cyanea* | Thraupidae | | 16 | 2000 |
| *Dubusia taeniata* | Thraupidae | | 2 | 2000-3000 |
| *Euphonia laniirostris* | Thraupidae | | 28 | 3000 |
| *Euphonia xanthogaster* | Thraupidae | | 87 | 1000 |
| *Hemispingus frontalis* | Thraupidae | | 4 | 1000 |
| *Hemispingus superciliaris* | Thraupidae | | 7 | 2000 |
| *Hemispingus verticalis* | Thraupidae | | 5 | 3000 |
| *Hemithraupis guira* | Thraupidae | | 11 | 2000 |
| *Iridophanes pulcherrima* | Thraupidae | | 8 | 1000 |
| *Iridosornis analis* | Thraupidae | | 15 | 1000 |
| *Iridosornis rufivertex* | Thraupidae | | 2 | 1000-2000 |
| *Pipraeida melanonota* | Thraupidae | | 2 | 3000 |
| *Piranga leucoptera* | Thraupidae | | 3 | 2000 |
| *Ramphocelus carbo* | Thraupidae | | 12 | 1000 |
| *Tachyphonus cristatus* | Thraupidae | | 8 | 1000 |
| *Tachyphonus rufus* | Thraupidae | | 2 | 1000 |
| *Tangara arthus* | Thraupidae | | 124 | 1000-2000 |
| *Tangara chilensis* | Thraupidae | | 531 | 1000 |
| *Tangara chrysotis* | Thraupidae | | 79 | 1000 |
| *Tangara cyanicollis* | Thraupidae | | 493 | 1000 |
| *Tangara gyrola* | Thraupidae | | 319 | 1000-2000 |
| *Tangara labradorides* | Thraupidae | | 47 | 1000 |
| *Tangara mexicana* | Thraupidae | | 2 | 2000 |
| *Tangara nigrocincta* | Thraupidae | | 25 | 1000 |
| *Tangara nigroviridis* | Thraupidae | | 196 | 1000 |
| *Tangara parzudakii* | Thraupidae | | 53 | 1000-2000 |
| *Tangara punctata* | Thraupidae | | 294 | 2000 |
| *Tangara ruficervix* | Thraupidae | | 1 | 1000 |
| *Tangara schrankii* | Thraupidae | | 293 | 1000 |
| *Tangara vassorii* | Thraupidae | | 310 | 1000 |
| *Tangara viridicollis* | Thraupidae | | 5 | 2000-3000 |
| *Tangara xanthocephala* | Thraupidae | | 84 | 1000-2000 |
| *Tangara xanthogastra* | Thraupidae | | 59 | 1000-2000 |
| *Tersina viridis* | Thraupidae | | 8 | 1000 |
| *Thlypopsis ornata* | Thraupidae | | 1 | 1000 |
| *Thraupis cyanocephala* | Thraupidae | | 162 | 3000 |
| *Thraupis episcopus* | Thraupidae | | 62 | 1000-2000 |
| *Thraupis palmarum* | Thraupidae | | 49 | 1000-2000 |
| *Volatinia jacarina* | Thraupidae | | 4 | 1000-2000 |
| Mt. Kilimanjaro |  | |  |  |
| *Coturnix coturnix* | Phasianidae | | 13 | 993 |
| *Francolinus sephaena* | Phasianidae | | 2 | 871 |
| *Francolinus squamatus* | Phasianidae | | 1 | 2800 |
| *Aplopelia larvata* | Columbidae | | 1 | 1800 |
| *Columba arquatrix* | Columbidae | | 62 | 1640–2970 |
| *Columba delegorguei* | Columbidae | | 4 | 2040 |
| *Streptopelia semitorquata* | Columbidae | | 1 | 950 |
| *Treron calva* | Columbidae | | 15 | 871–1640 |
| *Poicephalus rufiventris* | Psittacidae | | 7 | 950 |
| *Criniferoides leucogaster* | Musophagidae | | 31 | 950–1130 |
| *Tauraco hartlaubi* | Musophagidae | | 630 | 1250–3060 |
| *Colius striatus* | Coliidae | | 398 | 871–1750 |
| *Urocolius macrourus* | Coliidae | | 35 | 871–1130 |
| *Apaloderma vittatum* | Trogonidae | | 16 | 2040–2750 |
| *Bycanistes brevis* | Bucerotidae | | 210 | 1120–2040 |
| *Tockus deckeni* | Bucerotidae | | 19 | 950–1130 |
| *Tockus erythrorhynchus* | Bucerotidae | | 1 | 993 |
| *Tockus nasutus* | Bucerotidae | | 41 | 871–1130 |
| *Lybius leucocephalus* | Capitonidae | | 27 | 871–912 |
| *Pogoniulus leucomystax* | Capitonidae | | 49 | 1250–2040 |
| *Pogoniulus pusillus* | Capitonidae | | 12 | 871–950 |
| *Stactolaema leucotis* | Capitonidae | | 48 | 950–1620 |
| *Trachyphonus erythrocephalus* | Capitonidae | | 16 | 912–1130 |
| *Tricholaema lacrymosa* | Capitonidae | | 23 | 871–1560 |
| *Andropadus importunus* | Pycnonotidae | | 15 | 950–1130 |
| *Andropadus milanjensis* | Pycnonotidae | | 73 | 1640–2540 |
| *Andropadus nigriceps* | Pycnonotidae | | 1545 | 1650–3060 |
| *Phyllastrephus cabanisi* | Pycnonotidae | | 392 | 1650–2850 |
| *Phyllastrephus strepitans* | Pycnonotidae | | 60 | 950–2040 |
| *Pycnonotus barbatus* | Pycnonotidae | | 980 | 871–1750 |
| *Pseudoalcippe abyssinica* | Timaliidae | | 1 | 2040 |
| *Turdoides rubiginosa* | Timaliidae | | 34 | 993–1130 |
| *Cichladusa guttata* | Turdidae | | 9 | 871–1130 |
| *Irania gutturalis* | Turdidae | | 5 | 1130 |
| *Oenanthe oenanthe* | Turdidae | | 4 | 1130 |
| *Pogonocichla stellata* | Turdidae | | 15 | 1840–2800 |
| *Turdus olivaceus* | Turdidae | | 283 | 1620–3060 |
| *Zootheria piaggiae* | Turdidae | | 21 | 2120–2970 |
| *Bradypterus cinnamomeus* | Sylviidae | | 1 | 2040 |
| *Bradypterus lopezi* | Sylviidae | | 3 | 2040–2260 |
| *Cloroptera similis* | Sylviidae | | 1 | 2800 |
| *Hippolais languida* | Sylviidae | | 2 | 1130 |
| *Phylloscopus umbrovirens* | Sylviidae | | 5 | 1920–2260 |
| *Sylvia atricapilla* | Sylviidae | | 159 | 1130–1920 |
| *Sylvia borin* | Sylviidae | | 10 | 1020–1307 |
| *Sylvia communis* | Sylviidae | | 63 | 962–1310 |
| *Zosterops abyssinicus* | Zosteropidae | | 31 | 871–1120 |
| *Zosterops poliogaster* | Zosteropidae | | 1926 | 1620–3060 |
| *Batis molitor* | Platysteridae | | 2 | 1130–1640 |
| *Corvus albicollis* | Corvidae | | 4 | 2800–2940 |
| *Cinnyricinclus femoralis* | Sturnidae | | 66 | 2040–3060 |
| *Cinnyricinclus leucogaster* | Sturnidae | | 283 | 871–1560 |
| *Cinnyricinclus sharpii* | Sturnidae | | 58 | 1640–2940 |
| *Lamprotornis chalybaeus* | Sturnidae | | 51 | 871–1130 |
| *Lamprotornis corruscus* | Sturnidae | | 4 | 871 |
| *Lamprotornis superbus* | Sturnidae | | 24 | 950 |
| *Onychognathus morio* | Sturnidae | | 17 | 871–1360 |
| *Onychognathus walleri* | Sturnidae | | 290 | 1650–2940 |
| *Poeoptera kenricki* | Sturnidae | | 189 | 1300–2720 |
| *Petronia pyrgita* | Passeridae | | 30 | 871–1130 |
| *Anaplectes rubriceps* | Ploceidae | | 44 | 871–1560 |
| *Euplectes capensis* | Ploceidae | | 2 | 1130–1300 |
| *Ploceus intermedius* | Ploceidae | | 3 | 871–950 |
| *Ploceus nigricollis* | Ploceidae | | 3 | 912–1130 |
| *Ploceus ocularis* | Ploceidae | | 7 | 1120–2040 |
| *Ploceus rubiginosus* | Ploceidae | | 5 | 993 |
| *Ploceus velatus* | Ploceidae | | 1 | 871 |
| *Nigrita canicapilla* | Estrildidae | | 37 | 1620–2120 |
| *Linurgus olivaceus* | Fringillidae | | 66 | 1620–2970 |
| *Serinus citrinelloides* | Fringillidae | | 30 | 1620 |
| *Sernus mozambicus* | Fringillidae | | 18 | 871–1300 |
| *Emberiza flaviventris* | Emberizidae | | 3 | 871–1300 |
